# Mental illness stigma and its impact on help-seeking behavior among residents of the Eastern Region, Saudi Arabia: A cross-sectional study

**DOI:** 10.1371/journal.pone.0350860

**Published:** 2026-06-24

**Authors:** Yousif M. Elmosaad, Ziyad Alabdulqader, Mohammed Alhaddad, Mohammad Aatif, Ghazi I. Al Jowf

**Affiliations:** Department of Public Health, College of Applied Medical Sciences, King Faisal University, Al Ahsa, Saudi Arabia; ISSEP Kef: Universite de Jendouba Institut Superieur du Sport et de l’Education Physique du Kef, TUNISIA

## Abstract

**Background:**

Mental illnesses are documented as a public health concern; they are a leading cause of disability and poor quality of life among individuals. Most people with mental illness do not receive treatment due to many factors, including stigma, which significantly impacts the individual’s readiness to seek help.

**Methods:**

This is a cross-sectional study design conducted among individuals 18 years of age or older who lived in Al Ahsa. The data were collected through a structured questionnaire. A total of 1085 individuals participated. Descriptive statistics, chi-square test and binary logistic regression analysis were used to investigate the association and the predictors of help-seeking behavior.

**Results:**

Overall, the prevalence of mental illness-related stigma was 23.1%. There was a significant association between gender and some factors associated with mental illness stigma (p < 0.05). The proportion of individuals who might not seek help with mental illnesses due to stigma was 61.0%. Multivariate analysis indicates that the individuals who had mental illness-related stigma were more likely to avoid seeking help compared to the individuals who did not have stigma (B = 0.53, OR = 1.71, 95% CI: 1.25–2.34). The young adult group aged 30–41 years was found to be more likely to seek help (B = 0.47, OR = 1.80, 95% CI: 1.01–3.20) than the other age groups. However, gender, education, employment status, and marital status were not associated with help-seeking behavior (p > 0.05).

**Conclusion:**

The study indicates that the occurrences of stigma associated with mental illness was 23.1%. Approximately two-thirds of the participants in the study avoided seeking assistance for mental issues. Our multivariate analysis confirmed that the stigma related to mental illness is significantly linked to help-seeking behavior.Specifically, it was found that young adults are more likely to seek help compared to other age groups. Participants living in rural areas were less likely to seek help. These findings have significant implications for the formulation of interventions aimed at addressing mental illness stigma and promoting help-seeking behaviors.

## Introduction

Mental health influences the ability of individuals to make decisions, build relationships, and shape their environment [1–3]. It is an intrinsic component of overall health, intricately intertwined with physical health and behavior, and determined by a combination of biological, psychological, and social factors [4]. Several studies suggest that social and environmental factors increase the risk of mental illnesses among certain population groups [5–7].

Existing literature has shown that mental illness is associated with stigma which refers to negative attitudes, beliefs, stereotypes held by the public towards individuals with mental illness, leading to societal prejudice and discrimination against them [8,9]. Psychological frameworks describe stigma as encompassing stereotypes, labeling, isolation, status loss, prejudice, and discrimination [10]. Stigma can appear in various forms including public stigma and self- stigma, structural stigma and perceived stigma all of which influences the behaviors and willingness of individuals and families affected by mental illness to seek help [9]. Corrigan et al., reported that common stereotypes associated with individual with serious mental illness includes dangerousness, blame, and social incompetence [11]. Moreover, some previous studies have sought to conceptualize stereotypes that contribute self-stigma, which significantly influence help seeking behaviors, for example Ritsher *et al* [12] and others developed a five factor scale to assess self-stigma which includes alienation, stereotype endorsement, discrimination experience, social withdrawal and stigma resistance. This model was guided by different scale [13–15] used to examine whether individuals with mental illness are recognize the stereotypes related to mental illness. This conceptualization of stigma as progressive experience is critical in understanding how stigma can influence help seeking behavior the process begin with individuals’ awareness about societal stereotypes surrounding metal illness followed by agreement, where internalization these stereotypes as a facts it will lead to the application of these negative believe to oneself, resulting in server consequences such as reduce self-esteem and hesitant to seek help [14].

Recently, common mental illnesses have become highly prevalent in the population, affecting 25% of individuals across all regions around the world [16]. causing disability and accounts for 23% of all non-fatal burden [17]. Nevertheless, regional disparities arise due to differences in affluence and socioeconomic status. Global statistics from the World Health Organization (WHO) indicate that approximately one billion people are affected by mental illness. Notably, the prevalence of mental health conditions is significantly elevated in conflict-affected populations, reaching 22.1% [18]. In 2022, the USA data showed that about 23.1% of the adult population was living with a mental illness [19], while in Europe, it was found that 15.5% of young people experience a mental disorder [20]. The prevalence of mental disorders in the Gulf countries ranges from 15.6% to 35.5% [21]. In the UAE, according to Ghader *et al.,* it accounts for 19.9% of disease burden [22], Qatar 28% [23] and in KSA, specifically in Riyadh city, the prevalence was 28.5% among primary healthcare patients [24]. According to a recent study published in 2025, among the KSA population, it was reported that the lifetime prevalence of mental disorders is 34.2% [16].

Regarding health outcomes, mental illness, if left untreated, is significantly linked to negative results such as declining productivity, poor educational achievement, increased absenteeism, and higher turnover rates [25]. Similarly, untreated mental illness can lead to disability, substance abuse, homelessness, suicide, difficulties in maintaining friendships, and a reduced quality of life [20,26]. Moreover, failing to provide effective treatment for mental illness imposes a considerable economic burden on individuals and families and adversely affects society as a whole [27,28]. However, with appropriate and effective treatment, mental illness can be managed to substantially reduce 70 ~ 90% of these consequences [29].

Although mental illness is highly prevalent and effective treatments are well-established by a substantial body of evidence, more than 70% of individuals worldwide requiring mental health services do not have access to them [30,31]. In the Gulf region during the past 30 years, there has been an improvement in the availability of mental health services in hospitals delivered by healthcare professionals. For instance, in Bahrain, mental health services are provided free of charge by the Ministry of Health [32], and in Qatar, mental health services are being redefined and expanded. While in Kuwait and Oman, a review of mental health services found that it was still in a developmental stage [33].

In the context of the Kingdom of Saudi Arabia, the government has initiated Vision 2030, which includes the Health Sector Transformation Program. This program is designed to enhance the quality of life, ensure equitable access to healthcare services throughout the nation, and offer healthcare services at no cost (Vision 2030).  As part of this strategic initiative, substantial investments have been directed toward the development of mental health infrastructure. Currently, the country operates over 21 dedicated mental health hospitals, providing a total clinical capacity of 4,046 beds. In addition to 99 psychiatric clinics, and 14 specialized hospitals under construction [34].

Despite the availability of healthcare facilities dealing with mental illness, more than half of the psychiatric patients do not receive the required treatment or adequate social support [35]. Also, they may not receive suitable social support [36]. Thus, it is important to identify and address the factors preventing individuals from seeking mental health care and treatment. Previous research conducted in a different part of the globe suggests that many barriers prevent people from seeking mental health consultation. One of the main barriers is societal stigma, which comes from numerous sources, such as personal, social, and family beliefs and is frequently associated with reluctance or refusal to seek professional help [4,37].

In the Gulf countries, traditional cultural values, religious beliefs, and fear of social repercussions further exacerbate this issue and hinder seeking professional help [38,39]. Mental illness stigma exists at two broad levels: the community and personal levels. It’s a negative belief about mental illness that the community and/or an individual holds [40]. According to the MH Foundation, nearly 9 out of 10 people with a mental illness feel that stigma and discrimination negatively impact their lives [41].

Previous studies suggest that levels of stigma are the main stumbling block preventing patients from seeking mental health consultation and creating a gap between community, service, and treatment [38] and lead to different negative outcomes such as distress, low self-esteem, self-efficacy, treatment delay, treatment drop-out, exacerbating maladaptive behaviors, and reduced help-seeking behaviors [42]. Furthermore, other previous research reported that stigma has a major impact on help-seeking behaviors. Additionally, recent reviews of the influence of mental health-related stigma on help-seeking have reported that stigma is highly associated with decreased treatment adherence and seeking help [43,44]. Chandrasekara (2016) reported that there is evidence that prevailing societal norms surrounding mental illness would make them less likely to seek help [45].

The previous discussion illustrates the impact of social mental health-related stigma on help-seeking behavior and explains the importance of addressing stigma around mental illness to improve mental well-being. After reviewing the current situation of KSA regarding mental illness stigma and help-seeking behavior, the data showed that 22.3% of the Saudi population had at least one mental disorder, and only 13.9% of them obtained treatment [46]. 50% of those not seeking treatment hide their mental illness [47]. Moreover, another study reported that 36.5% of the general population could speak to any person with a mental illness [48]. Based on the previous studies conducted in the Saudi context have reported the prevalence of mental illness and highlighted that only a small proportion of affected individuals receive treatment despite the availability of mental health services. However, these studies have not clearly identified the underlying reasons for low treatment uptake or the lack of social acceptance toward people with mental illness. This study contributes existing literature by examining the relationship between stigma and help-seeking behavior, offering a deeper understanding of the determinants of stigma and help seeking.

Nevertheless, this demonstrates that there is a need for conducting a community based survey in the Eastern Region, to estimate the prevalence of mental illness stigma and exploring the association of the stigma with help-seeking behavior in the general population. Additionally, the survey aims to estimate the proportion of individuals who might not seek help with mental illnesses for fear of perceived social stigma. Furthermore, the study hypothesized that individuals who have a stigma associated with mental illness are less likely to seek help for their own mental illness. Finally, we provide some recommendations to policymakers to develop effective stigma reduction strategies.

## Methods

### Study design and setting

A cross-sectional study design conducted in Al Ahsa province, located in the eastern part of Saudi Arabia, Data were collected during the period from October 27^th^ to November 3^rd^, 2024 and data analysis was conducted in May 2025. The total area is 410,713 km^2^, which includes three administrative areas: Al-Hofuf, Al-Mubaraz, and Al-Qura [49].

### Study population

The total population of the province is 1,300,000, of which 1,095,344 are aged 18 years or older, among them 42.0% of them living in Al-Hofuf, 38.0% Al Mubarraz and 20% from Al-Qura (Al Rajeh et al 2022) [50], with a total population growth of 2.0% according to the Central Authority for Statistics. The entire population of Al Ahsa, 18 years or older, who lived in Al Ahsa province, of all genders, was considered eligible to participate in the study.

### Inclusion criteria

The inclusion criteria were participants aged 18 years or older who lived in Al Ahsa during the reference period, with the ability to competently comprehend written Arabic and/or English texts. The exclusion criteria were participants who were unwilling to participate in the study or who lived outside Al Ahsa province.

### Sample techniques

A multi-stage sampling method was used to draw a representative sample of adults 18 years or older who resided in the Al Ahsa administrative areas of Al-Hofuf, Al-Mubaraz, and Al-Qura. The required study sample size was 767, calculated using Epi Info software with an absolute precision of 5% and at a 95% confidence interval and 2x design effect. To draw a representative sample, the total sample (767) was proportionally allocated according to the total population of the three administrative areas: Al-Hofuf accounts for 322 (42%) participants of the total sample, followed by Al-Mubaraz with (291) 38%, and Al-Qura with 153 (20%). After distributing the study sample by administrative areas, the total number of residential areas was identified: Al-Hofuf had 12 residential areas, Al-Mubaraz had 4 residential areas, and there were 14 villages. Out of these, 50% were randomly selected and proportionately distributed, 6 from Al-Hofuf, 2 from Al-Mubaraz, and 7 from Al-Qura.

To determine the number of study participants for each selected residential area, proportional allocation based on the population size of each area was used. Participants within each residential area, were then selected from the residents’ lists using a fixed interval value. The interval value is calculated by dividing the required sample size for that residential area by its total population.After participants were selected, they were contacted through the available means of communication such as: social media platforms (WhatsApp, Telegram, Snapchat), and mobile phones to schedule an interview for completing the questionnaire. The data collection was collected by six public Health students who were specially trained to collect responses from participant. A total of 1085 responses were collected, exceeding the minimum required sample size of 767.Among the eligible individuals who were contacted, six declined to participate, resulting in a non-response rate of 0.6%. This higher number was due to the higher-than-expected participation in Al hofuf and Al Qura. All responses were verified for completeness and validity and were included in the final analysis. Study participants’ detailed information is presented in Table 1.

**Table 1:** Socio-demographic characteristics of the study participants (n = 1085).

Variables	Responses	Count	Percentage
Age in years	• 18 – 29 years	320	29.5%
	• 30 – 41 years	170	15.7%
	• 42 – 53 years	256	23.6%
	• 54 – 65 years	290	26.7%
	• Older than 65 years	49	4.5%
Gender	• Male	511	47.1%
	• Female	574	52.9%
Location of Residence in Al Ahsa	• Al-Hofuf	682	62.9%
	• Al-Mubarraz	221	20.4%
	• Al-Qura	182	16.8%
Education Level	• No formal education	4	0.4%
	• High school or lower	230	21.2%
	• College level or equivalent	750	69.1%
	• Master or higher	101	9.3%
Employment Status	• Student	226	20.8%
	• Employed	392	36.1%
	• Unemployed	146	13.5%
	• Retired	321	29.6%
Marital Status	• Single	305	28.1%
	• Married	733	67.6%
	• Divorced	23	2.1%
	• Widowed	24	2.2%
Experience dealing with an individual with mental illness	• Yes	412	38.0%
	• No	673	62.0%

### Data collection

Before starting data collection, the study protocol was approved by the college’s research committee and the KFU Deanship of Scientific Research Ethics Committee, bearing the approval number (KFU-REC-2024-OCT-ETHICS2653). The study was conducted in accordance with the local legislation and institutional requirements. The participants provided their written informed consent to participate in this study.The survey was conducted during the period from October 27^th^ to November 3^rd^, 2024. Data was collected using an anonymous, structured questionnaire designed in both English and Arabic designed by researchers; it will take 5–10 minutes to complete. The study participants were interviewed after providing formal consent to participate in the study. They were also informed about the study objectives, and the confidentiality and privacy of the data gathered were maintained. We ensured data accuracy, integrity, missing responses and facilitated the data entry process. The questionnaire was designed using Google Forms. Data collectors share the link with participants at the beginning of the interview all responses were recorded under their guidance.

### Instrument and measurement

The questionnaire was developed based on the key concepts derived from “ Link’s Devaluation–Discrimination Scale” [13], and” Self-Stigma of Mental Illness Scale” [51], specifically related to the variables concerning to stigma and help-seeking in addressing psychological challenges. Some items rephrased to align with the linguistic and educational level of the study population, as well as to minimize response time. The content validity was ensured through a combination of rigorous literature review, consultation with public health specialist and focus group discussions. Based on their feedback, certain items were modified or removed to improve clarity and relevance based on their feedback. Moreover, a factor analysis was conducted, of the five item stigma scale (N = 1085) using principal component analysis with Varimax rotation. The KMO measure was 0.491 and Bartlett’s test was significant (p < 0.001) supporting factorability. Communalities ranged from 0.27 to 0.64, indicating adequate item contribution. Additionally, to assess the reliability of the questionnaire, a pilot study was conducted on a random sample from the study population. Cronbach’s alpha was calculated. The results reveal the questionnaire demonstrate high validity, with a Cronbach’s alpha equal (0. 83).

The data collection tool consisted of four parts. Part one included variables related to the participant’s socio-demographic characteristics, such as age, gender, residence location, education level, employment status, marital status, and experiences. Furthermore, there were inquiries regarding participants’ interactions with individuals suffering from mental illness. Part two of the questionnaire comprised five questions used to assess the prevalence of mental illness-related stigma. Part three consisted of one question with multiple responses, used to identify factors associated with mental illness stigma. The final part included four questions to determine the proportion of individuals who might not seek help for mental illnesses due to stigma.

We assessed the prevalence of mental illness-related stigma through 5 questions. Each question had different response options. The first question relates to avoiding interaction with someone you know who is suspected of or suffering from a mental illness. For this question, a response of “frequently” was given 2 points; “occasionally” was given one point, and “never” was given zero points. The second question assesses the willingness to interact with someone with a mental illness. For this question, a response of “Not receptive” was given 2 points, “Neutral” was given one point, and “Receptive” was given zero points. The third question measures agreement with the statement that “mental illnesses are caused by weak personality”. For this question, a response of “Agree” was given 2 points, “Neutral” was given one point, and “Disagree” was given zero points. The fourth question pertains to their experiences or observations of an individual within their community being subjected to mistreatment (such as bullying, insults, or physical violence) due to their mental illness. For this question, a response of “Frequently” was given 2 points, “Occasionally” was given one point and “Never’ was given zero points. The final question assesses the impact of the respondent’s perception of mental illness on their willingness to support mentally ill individuals in their home. For this question, a response of “Increases support” was given zero points, “No effect” was given one point and “Decreases support” was given 2 points. An overall score of mental illness-related stigma was calculated by adding up the score for each of the 5 questions, and the maximum total score was 10, with a range from 0 to 10 points. The overall mental illness-related stigma was dichotomized into two categories (do not have stigma and have stigma) based on their cumulative grade. A score below the mean (< 5) indicates no mental illness related stigma, whereas a score equal to or above the mean (≥ 5) indicates the presence of mental illness related stigma [49]. We identify the factors linked to mental illness stigma by one question with multiple responses (10 responses), and the participants choose the factors they consider related to mental illness stigma.

We determined the proportion of individuals who might not seek help with mental illnesses due to the stigma through two (Yes/ No) questions. For these questions, a response of (No) was given one point, and a response of (Yes) was given zero points. However, for Likert scale questions, a response of high possibility was given 2 points, a low possibility was given one point, and improbable was given zero points. The maximum total score was 6, with a range from 0 to 6 points. A cutoff point was identified based on the total range of values, where a score of less than 3 indicates seeking help and a score of 3 or higher indicates being deterred from seeking help.

### Statistical analysis

Before the statistical analysis, the dataset was migrated to MS Excel to be thoroughly cleaned for inconsistencies. Then it was coded according to the operational definitions and the categories as mentioned in the Instrument and Measurement part. Cleaned data were analyzed by using the Statistical Package of Social Science (SPSS) version 25.Descriptive statistics were used to determine the frequencies and percentages of the socio-demographic characteristics, such as age, gender, location of residence, education level, employment status, marital status and experiences, while continuous variables were presented in the form of mean and standard deviation (SD). A multivariate binary logistic regression analysis was used to determine the effect of independent variables on the association between mental illness-related stigma and help-seeking behaviors. Crude and adjusted odds ratios (OR/AOR) were presented with a 95% CI. A p-value less than 0.05 were considered statistically significant. The raw datasheet is provided as supporting (S1 Data: sav file and S2 Data: excel sheet).

## The Results

The socio-demographic characteristics of study participants are presented in Table 1. All study participants were 18 years or above (1085); of them, 511 (47.1%) were female and 574 (52.9%) were male. More than half of the participants were older than 42 years, 595 (54.8%). Based on the location, the majority of study participants were from Al-Hofuf 682 (62.9%), followed by Al-Mubarraz 221 (20.4%) and Al-Qura 182 (16.8%). About 750 (69.1%) of the participants had a college education level or equivalent. The majority of participants were married, 733 (67.6%), and more than one third of the study participants were employed, 391 (36.0%), followed by retired participants, 321 (29.6%), and students represented 266 (20.8%) of the study participants. Over one-third of the participants, 673 (62.0%), do not have experience dealing with someone suspected or suffering from a mental illness.

The prevalence of mental illness-related stigma is presented in Table 2. The results illustrate that 166 (15.3%) of the participants frequently avoided interactions with someone known or suspected to be suffering from mental illness, and 139 (12.8%) of them did not openly interact with mentally ill individuals. Likewise, 182(16.8%) of the participants agreed that mental illnesses are caused by weak personalities, and 232 (21.4%) of the participants frequently witnessed someone treated harshly due to mental illness. Only 48 participants, representing 4.4%, believed that mental illness affects their willingness to assist individuals with mental health issues. The overall prevalence of stigma related to mental illness is 251, accounting for 23.1%, with 12.9% among males and 10.2% among females. According to the chi-square test, there exists a significant correlation between gender and the prevalence of stigma associated with mental illness (p < 0.05).

**Table 2:** Prevalence of mental illness-related stigma (n = 1085).

Variables	Responses	Frequency	Percentage	95% CI
Avoidance of interaction with a known or suspected mental illness case.	Never	387	35.7%	32.8–38.6
	Occasionally	532	49.0%	46.0–52.0
	Frequently	166	15.3%	13.2–17.4
Open to interactions with mentally ill individuals.	Receptive	474	43.7%	40.7–46.7
	Neutral	472	43.5%	40.6–46.4
	Not receptive	139	12.8%	10.8–14.8
Agreement with the following statement: “Mental illnesses are caused by weak personalities”.	Disagree	640	59.0%	56.1–61.9
	Neutral	263	24.2%	21.7–26.7
	Agree	182	16.8%	14.6–19.0
Witnessed someone being treated harshly due to mental illness.	Never	416	38.3%	35.4–41.2
	Occasionally	437	40.3%	37.4–43.2
	Frequently	232	21.4%	19.0–23.8
The influence of mental illness perception on support for mentally ill individuals.	Increased support	826	76.1%	73.6–78.6
	No effect	211	19.4%	17.0–21.8
	Decrease support	48	4.4%	3.2–5.6
Overall prevalence of stigma	No stigma	834	76.9%	74.4–79.4
	Has stigma	251	23.1%	20.6–25.6
	251(23.1%) overall prevalence of mental illness-related stigma, (12.9%) among males and (10.2%) among females*.			

*p-value significant at level ≤0.05

To determine the association between mental illness stigma-associated factors and variations based on participants’ gender, the chi-square test was used. As shown in Table 3, there was a significant association between gender and some factors associated with mental illness stigma (p < 0.05). Male participants specifically recognized friends, peer influence and school or university environment as factors associated with mental illness stigma (55.1%, 52.3% and 52.2% respectively). However, female participants specifically recognized family beliefs as a factor associated with mental illness stigma (55.1%).While other factors, such as workplace policies, health awareness campaigns, social media platforms, quality of mental care, and personal mental health experiences, are not associated with the participants’ gender (p > 0.05). However, these factors are not associated with any gender. All genders report the following factors associated with mental illness stigma: quality of mental care at 473 (43.6%), social media platforms at 465 (42.9%), school or university environments at 383 (35.3%), health awareness campaigns at 373 (34.4%), personal mental health experiences at 282 (26.0%), and workplace policies at 250 (23.0%).

**Table 3:** Factors associated with mental illness stigma variations based on participants’ gender (n = 1085).

Factors linked to mental illness stigma	No. / %	Male		Female		x^2^	p-value *
		No.	%	No.	%		
Family beliefs	660 (60.8%)	299	45.3	361	54.7	4.17	0.050
Friends and peer influence	526 (48.5%)	290	55.1	236	44.9	26.46	0.001
Culture and religion	398(36.7%)	208	52.3	190	47.7	6.73	0.006
Workplace policies	250 (23.0%)	126	50.4	124	49.6	1.42	0.131
School or university environment	383(35.3%)	200	52.2	183	47.8	6.23	0.007
Quality of mental health care	473(43.6%)	224	47.4	249	52.6	0.23	0.464
Health awareness campaigns and initiatives	373(34.4%)	185	49.6	188	50.4	1.43	0.129
Social media platforms	465(42.9%)	227	48.8	238	51.2	0.97	0.178
Personal mental health experiences	282 (26.0%)	135	47.9	147	52.1	0.92	0.407

*p-value significant at level ≤0.05

The results presented in Table 4 show that 771(71.1%) of the participants perceive that there is a negative stigma surrounding mental illnesses in their communities that prevents them from seeking help. Furthermore, more than one quarter of the participants 277 (, 25.5%) of the participants avoided seeking help for a mental illness due to fear of being judged or stigmatized, and 230 (21.2%) of the participants indicated a high probability of avoiding professional help due to fear of stigma. Additionally, 367 (33.8%) of the participants indicated a high likelihood of avoiding discussing mental health issues with relatives and friends due to fear of stigma. Overall, 662 (61.0%) of the study participants were deterred from seeking help for mental illnesses due to stigma, 320(48.3%) of them being male and 342(51.7%) of them being female. According to the chi-square test, a significant relationship exists between gender and reluctance to seek assistance for mental illnesses due to stigma (p < 0.05).

**Table 4:** Proportion of individuals who might not seek help with mental illnesses due to stigma.

Variables	Response	Frequency	Percentage	95% CI
They believe that, there is a negative stigma in their community that prevents them from seeking help for mental illnesses.	No	314	28.9	26.2 – 31.6
	Yes	771	71.1	68.3–73.7
Avoidance of seeking help for a mental illness due to fear of being judged or stigmatized.	No	808	74.5	71.1–77.1
	Yes	277	25.5	22.9–28.1
Avoidance of seeking professional help due to fear of stigma.	Improbable	338	31.2	28.4–34.0
	Low probability	517	47.6	44.6–50.6
	High probability	230	21.2	18.8–23.6
Avoidance of discussing mental health issues with relatives and friends due to fear of stigma	Improbable	212	19.5	17.1–21.9
	Low probability	506	46.6	43.6–49.6
	High probability	367	33.8	31.0–36.6
Overall proportion of seeking help with mental illnesses	Seeking help	423	39.0	36.1–41.9
	Deters individuals from seeking help	662	61.0	58.1–63.9
	Overall, 662 (61.0%) of the study participants were deterred from seeking help for mental illnesses due to stigma, with 320(48.3%) of them being male and 342 (51.7%) of them being female*.			

*p-value significant at level ≤0.05

**Fig 1:**
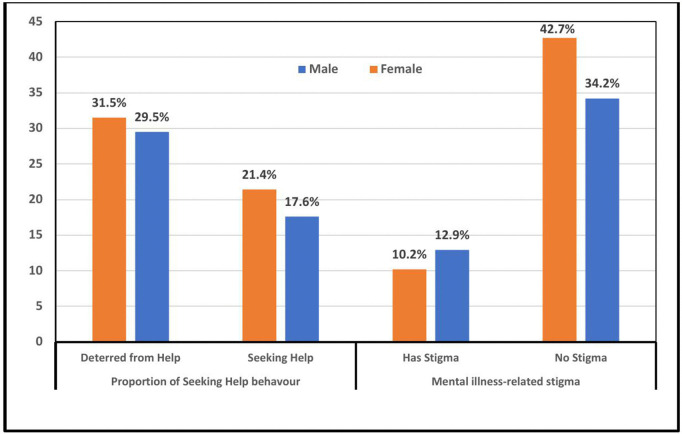
The association between the prevalence of mental illness-related stigma and help-seeking behavior based on participants’ gender.

The results of the chi-square test indicated a notable association between the occurrences of stigma related to mental illness (p < 0.05). However, no significant differences were found in the proportion of help-seeking behavior across genders (p > 0.05). Also, there was a significant association between the prevalence of mental illness-related stigma and help-seeking behavior. (p < 0.05) Fig 1.

Our multivariate analysis shows that stigma related to mental illness is significantly linked to help-seeking behavior. Specifically, individuals with mental illness-related stigma are more likely to avoid seeking help due to stigma compared to those without stigma, (B = 0.53, OR = 1.71, 95% CI: 1.25–2.34). Additionally, age and residence are important factors because they are significantly associated with help-seeking behavior. Notably, the young adult group aged 30−41 years was more likely to seek help (B = 0.47, OR = 1.80, 95% CI: 1.01–3.20) than other age groups. Conversely, residents of Al-Qura were less likely to seek help (B = −0.48, OR = 0.62, 95% CI: 0.44–0.88) compared to those living in Al-Hofuf. However, experience in dealing with individuals with mental illness was not associated with help-seeking behavior (B = 0.22, OR = 1.25, 95% CI: 0.96–1.62) compared to those without such experience (p > 0.05). Similarly, gender, education, employment status, and marital status showed no significant association with help-seeking behavior (p > 0.05) Table 5.

**Table 5:** Predictors of help-seeking behavior among the population living in Al Ahsa, Saudi Arabia (n = 1085).

Predictors		Unadjusted model			Adjusted model		
		B	OR (95% CI)	*p*-value	B	OR (95% CI)	*p*-value
**Prevalence of** mental illness-related stigma	Individuals with no Stigma	R.					
	Individuals who has Stigma	0.54	1.72(1.27-2.33)*	0.001	0.54	1.71(1.25-2.34)*	0.001
Age in years	18-29	R.					
	30-41	0.47	1.59(1.06-2.39)*	0.025	0.59	1.8(1.00-3.2)*	0.046
	42-53	-0.36	0.96(0.69-1.35)	0.833	0.20	1.22(0.71-2.13)	0.48
	54-65	-0.37	0.69(0.50-0.95) *	0.4230.023	-0.04	0.96(0.51-1.82)	0.900
	>65	-0.13	0.88(0.478-1.63)	0.687	0.31	1.37(0.57-3.28)	0.483
Gender	Male	R					
	Female	-0.13	0.88(0.69-1.12)	0.305	-0.11	0.90(0.68 -1.20)	0.462
Place of residence	Al-Hofuf						
	Al-Mubarraz	-0.23	0.79(0.58-1.08)	0.149	-0.23	0.8(0.58-1.1)	0.161
	Al-Qura	-0.41	0.66(0.48-0.92)*	0.015	-0.48	0.62(0.44-0.88)*	0.007
Education Level	No Formal Education	R					
	School Level	0.73	0.48(0.05-4.71)	0.530	-0.66	0.52(0.05-5.19)	0.576
	Collage Level or Equivalent	-0.64	0.53(0.05-5.10)	0.581	-0.66	0.52(0.05-5.19)	0.576
	Higher Education	-0.58	0.56(0.06-5.57)	0.620	-0.87	0.42(0.04-4.34)	0.466
Employment Status	Student	R					
	Employed	0.16	1.17 (0.832-1.64)	0.530	0.25	1.29(0.75 -2.22)	0.362
	Unemployed	0.05	1.1 (0.68-1.61)	0.581	0.261	1.30(0.70-2.41)	0.41
	Retired	-0.33	0.72(0.51-1.02)	0.620	0.07	1.07(0.54-2.13)	0.84
Marital Statues	Single	R					
	Married	-0.24	0.79(0.59-1.04)	0.090	-0.42	0.66 (0.4-1.09)	0.103
	Divorced	-0.19	0.83(0.35-1.98)	0.672	-0.39	1.68 (0.253 -1.83	0.445
	Widowed	-0.63	0.53(0.23-1.23)	0.139	-0.67	0.51 (0.193 -1.35)	0.177
Experience	Yes						
	No	0.21	1.23(0.95-1.59)	0.106	0.22	1.25 (0.96-1.62)	0.101

*Dependent variable: Help-Seeking Behavior.

## Discussion

Mental health disorders are recognized as a significant public health issue globally; they rank among the primary contributors to disability, substance dependence, homelessness, suicide, and diminished quality of life for individuals [20,24,28]. In Europe and the USA, 52–74% of people with mental illness did not receive treatment due to stigma, which significantly impacted individuals’ readiness to seek help [30,44,52]. Estimating the prevalence and impact of stigma on help-seeking behavior and identifying factors associated with mental illness stigma is essential for improving population mental health outcomes [52]. Based on this focus, our study aimed to estimate the prevalence of mental illness stigma assess its impact on help-seeking behavior and to estimate the proportion of those who might not seek help due to stigma.

### Prevalence of stigma-related mental illnesses

Our primary findings can be examined from various perspectives, including the widespread stigma associated with mental illness. Our research results revealed certain behaviors linked to mental illness stigma, such as reluctance to provide support, avoidance, and a lack of open interaction with individuals known or suspected to have mental illnesses; all of these behaviors contribute to the overall prevalence of mental illness-related stigma, which was recorded at 23.1%. The prevalence was found to be lower in developed nations like the United States [53] compared to developing countries such as Nigeria (41.5%) [54] Qatar (41.8%) [55] and China (49.5%) [56]. (These comparisons should be interpreted cautiously due to the differences in instruments, sampling and cultural context). This evidence documented that the prevalence of stigma-related mental illness varies across countries, which could be the result of the differences in the level of public awareness, cultures, religious values, levels of social support, level of development and accessibility to preventive services.

Interestingly, in terms of the prevalence of mental illness, stigma was lower in KSA than in developed and developing countries. This might be due to the KSA witnessing immense transformation in education, social and health sectors, in addition to an increase in public awareness, electronic health care services and universal health care coverage that can improve access and quality of care.

### Gender-based differences in prevalence of mental illness stigma

On the other hand, the stigma is significantly associated with gender; the prevalence was higher among males than among females. This finding is inconsistent with a study conducted in India by Kiesza et al., who reported that mental illness stigma among women is greater than among men [57,58], while consistent with other studies, which highlighted that mental illness stigma among males is higher than among females [59]. Another study reported there is no difference at all in the prevalence of stigma among all genders [25]. (These comparisons should be interpreted cautiously due to the differences in instruments, sampling and cultural context). Based on these findings, we observed that males and females experienced stigma, though the prevalence of stigma-related mental illness varies among them, which may allow us to suggest that all genders should receive mental health services to lower stigma and develop positive attitudes towards mental illness.

In terms of the factors linked to mental illness stigma, the study’s findings indicates that males and females concurred on several influences, including family beliefs, peer and friend impact, the educational environment, social media platforms, health awareness initiatives, and personal experiences, as contributing factors to mental illness stigma. This observation aligns with some of the factors identified by NASM in 2016. Similarly, another research study conducted in the UAE highlighted the significance of traditional culture, family, and religious values [60]. Based on the preceding discussion, it is evident that there is a lack of consensus among researchers regarding the factors related to mental illness stigma, as they approach the topic from various perspectives and angles.

### Help-seeking behavior and associated factors

Significantly, the examination of the variables related to help-seeking behavior is noteworthy. Our research indicates that approximately two-thirds of the participants in the study refrained from seeking assistance for mental illnesses due to the negative stigma associated with mental health in their communities, apprehension about being judged or stigmatized, and a reluctance to discuss their situation with family and friends, particularly among males compared to females. Numerous prior studies have addressed the barriers to help-seeking in various regions around the world.They reported that not seeking help for mental illnesses is influenced by a range of factors such as fear of being judged, family beliefs, fear of losing face, labelling, status loss, separation and discrimination [57,61,62]. It is also worth noting that recent reviews and meta‐analyses have confirmed the association and correlation between mental illness-related stigma and help‐seeking [63–65]. On the other hand, the study reported that stigma significantly associated with lower help-seeking behavior and treatment dropout [4,66]. These findings support the notion that providing mental health services, addressing mental illness stigma, promoting evidence-based treatment and incorporating religious and social values increase the likelihood of help-seeking behavior.

### Predictors of help-seeking behavior

Our multivariate analysis confirmed that the mental illness-related stigma is significantly associated with help-seeking behavior; in other words, individuals who perceive mental illness-related stigma are more likely to avoid seeking help. This is consistent with findings of studies conducted in China [67] and India [57]. Precisely, the young adult group of 30–41-year-olds was determined to be more likely to seek help than other age groups. These findings align with previous research within the Saudi context suggesting that younger Saudis tend to be more openness to help‑seeking compared to older generations [68]. While it’s in contrast to Vanheusden *et al*., who reported that young adults are less likely to seek help for mental health problems [69].

Generally this reflect the generational shift young adults, are increasingly inclined to seek assistance, The younger generation has become more open to mental health discussions as a result of both local and global awareness campaigns and social media influence, which contributed in their healthy literacy and enhanced their understanding of mental illnesses. Among young adults, females have increasingly engaged with mental health services due to the societal changes, greater awareness and a greater tendency toward emotionally expressive to seek support when facing challenges. However, males, particularly older individuals, may face stigma and cultural barriers that discourage seeking help [70]. The culture expectation of masculinity also discourage emotional expression and help seeking behaviors, as males are expected to handle their problem independently and without external assistance. This may scaling up stigma around mental illnesses and further discouraging individuals from seeking help from the appropriate sources [71]. Conversely, residents of Al-Qura were found to be less inclined to seek help. This may be due to individuals living in rural areas being more likely to adhere to their cultural values and beliefs. Nevertheless, other sociodemographic factors such as gender, education, employment status, and marital status did not show any association with help-seeking behavior. These findings align with those of a prior study conducted in China [72] and are consistent with the findings of Clark et al. in 2020, which indicated that females are more likely to seek help compared to males [73].

Taken together, these findings suggest that efforts to reduce mental-illness stigma and promote help-seeking should consider ageand gender-specific sociocultural factors.Targeted interventions for younger adults may leverage their openness and access to information, while strategies aimed for men and residents of certain regions should address cultural barriers and societal expectations that inhibit help-seeking within Saudi community.

## Conclusion and Recommendations

Our study reveals that the prevalence of mental illness-related stigma was lower as compared with that reported neighboring countries such as Qatar. Nevertheless, around two-thirds of the study participants avoided seeking help for mental illnesses. Though all gender agreed upon family beliefs, friends and peer influence, school or university environment, social media platform, health awareness campaigns and personal experiences respectively are factors associated with mental illness stigma. Our multivariate analysis confirmed that the mental illness-related stigma is significantly associated with help-seeking behavior. Precisely, young adults were determined to be more likely to seek help than other age groups. Participants resident in rural areas were less likely to seek help. These results carry significant implications for the formulation of interventions aimed at addressing mental illness stigma and promoting help-seeking behavior. It is essential to set policies, create a supportive environment, promote access to mental health care, offer evidence-based treatment and have a positive impact on help-seeking outcomes. However, further applied research is necessary to ensure a beneficial impact on help-seeking outcomes.

### Study limitation

There are many limitations that need to be taken into consideration. First: the assessment of prevalence and help-seeking behavior depends on participants’ self-reports, which may be subject to recall bias, social desirability bias, and the study subjected to non-response bias, which may limit the accuracy and generalizability of the finding to mitigate these potential biases, we emphasized anonymous and confidential data collection and structured questions to reduce judgmental framing. Second: content validity: In absence of conducting factor analysis, content validity was assessed based on rigorous literature review, expert consultation, focus group discussions also guided by the general concept of the stigma scale “Link’s Devaluation–Discrimination Scale” [13], and” Self-Stigma of Mental Illness Scale”, which may affect the generalizability of the results. Third: this research faces limitations regarding the diversity of the geographic regions represented in the sample. Fourth, the cross-sectional study design may preclude any causal relationship from being established between influencing factors and formal help-seeking. Future studies should consider these limitations, thus increasing the research results’ applicability and utilization.

**Informed Consent Statement:** Informed consent was obtained from the study participants, who were briefed about the purpose of the study, the confidentiality and privacy of data gathered, and assurance that their information would only be used for the intended purposes of this study.

## Supporting information

S1 Data Dataset file sav format (S1_sav_Supplementry file). (SAV)

S2 Data Dataset file excel format (S2_data file_excel). (XLSX)
