# Supplementary material for: Mental illness stigma and its impact on help-seeking behavior among residents of the Eastern Region, Saudi Arabia: A cross-sectional study
Source: PLoS One. 2026 Jun 24;21(6):e0350860. doi: 10.1371/journal.pone.0350860 (PMC13293441; doi:10.1371/journal.pone.0350860)
Supplement: Attachment — Submitted filename: Second_Response_to_Authors_Stigma_PLOS.pdf [file pone.0350860.s006.pdf]

Re: Second Review - Response to Authors' Replies

Manuscript: Mental Illness Stigma and its Impact on Help-Seeking Behavior among Residents of the Eastern Region, Saudi Arabia: A Cross-Sectional Study

Journal: PLOS ONE

Dear Editor,

I thank the authors for their revised manuscript and their point-by-point responses to the first round of comments. I acknowledge that several concerns have been adequately addressed, including expanding the theoretical framework in the introduction (Corrigan & Watson; Link & Phelan), addressing the socio-cultural interpretation of gender and age differences in help-seeking, and revising the language and formatting.

However, several substantive methodological issues remain insufficiently resolved. I outline these below, distinguishing between points that I consider satisfactorily addressed, those that require minor revision, and those that represent major outstanding concerns.

1. Points Considered Satisfactorily Addressed

The following points from the first round have been addressed to a satisfactory degree and require no further revision:

• 3.4 (multicollinearity check), 5.1–5.3 (language corrections), 6.1 (ethical approval), 7.1 (STROBE alignment), 7.2 (table corrections), 8.1 (theoretical grounding), 11 (causation vs. correlation language), 15–20 (minor corrections).

2. Major Outstanding Concerns

Point 3.2 and 9.1 and 9.2 - Instrument Validation (MAJOR, UNRESOLVED)

Original concern: The self-developed stigma and help-seeking scales lack validation against recognized instruments. Factor analysis and additional reliability indices were requested.

The authors' response acknowledges the absence of formal validation but argues that conceptual alignment with Link's Devaluation–Discrimination Scale and the Self-Stigma of Mental Illness Scale is sufficient. This is not acceptable for a PLOS ONE submission. Conceptual alignment during item generation is a preliminary step, not a substitute for psychometric validation.

Specifically:

(a) The claim that the questionnaire is "not a novel scale" and therefore does not require factor analysis is incorrect. Any self-developed instrument, regardless of its conceptual origins, requires structural validation particularly when used to produce prevalence estimates that form the primary outcome of the study.

(b) Cronbach's alpha (0.83) measures internal consistency but provides no evidence of construct validity, dimensionality, or criterion-related validity.

(c) The revised manuscript should either: (i) report an exploratory or confirmatory factor analysis to establish the dimensional structure of the instrument; or (ii) include an explicit acknowledgment in the Limitations section that the absence of formal psychometric validation is a significant constraint on the generalizability and comparability of findings. This limitation must be discussed substantively, not simply mentioned in passing.

Point 3.5 and 10.1 - Dichotomization Thresholds (MAJOR, PARTIALLY RESOLVED)

Original concern: Dichotomization thresholds are arbitrary. Reanalysis using continuous or ordinal scales was recommended.

The authors justify using the mean as a cutoff by citing two previous studies (Lapham et al., 2022; Hadera et al., 2019). This justification remains weak. Neither reference appears to use a comparable instrument or study design, and citing precedent for a practice does not resolve its methodological limitations.

Furthermore, note that the response document itself contains an internal contradiction: under Point 3.5, it states both 'Mean score ≥ 5 indicates no mental illness-related stigma' AND 'indicates presence of mental illness-related stigma', these are mutually exclusive and suggest the revision was not reviewed carefully.

Furthermore, the Methods section (p. 12) contains another logical contradiction in the definition of the help-seeking cutoff that was not present in the response letter but appears in the revised manuscript: *"a score of less than 3 indicates seeking help and a score <3 indicates being deterred from seeking help"*. Both conditions use the same threshold (<3) to define two mutually exclusive outcomes, which is logically impossible and renders the operationalization of the outcome variable unintelligible.

The authors should at minimum: (i) correct the internal contradictions noted above in both the response document and the manuscript; (ii) provide a more substantive theoretical or empirical rationale for the specific cutoff of 5 (not simply that the mean was used); (iii) acknowledge in the Limitations section that dichotomization leads to loss of information and may inflate or deflate prevalence estimates depending on the distribution of scores.

Point 6.2 and 13 - Data Availability (UNRESOLVED, POLICY VIOLATION)

This issue was raised in the first round of review and remains unresolved. The submission form contains a direct contradiction: the authors selected "Yes - all data are fully available without restriction" but then wrote in the text box below: *"supplementary data can be provided on request"*. These two statements are mutually exclusive. Additionally, the Data Availability statement in the manuscript (p. 13) states that data are "available as supplementary file" without providing any repository link, DOI, or accession number.

PLOS ONE's data availability policy is unambiguous: data must be fully and unconditionally available at the time of publication, without requiring readers to contact the authors. Stating that data are available "on request" or "as a supplementary file" without a retrievable location does not meet this requirement.

To comply, the authors must take one of the following actions before resubmission:

1. Deposit the full de-identified dataset in a recognized public repository (e.g., Figshare, Zenodo, OSF, or Dryad), obtain a DOI, and include it in the Data Availability Statement; or
2. Upload the dataset as a formal supplementary file through the journal submission system, ensuring it is publicly accessible upon publication - not upon request.

The Data Availability Statement in the manuscript must then be updated to reflect exactly where and how the data can be accessed, with a direct link or DOI. Failure to resolve this point may result in rejection on editorial policy grounds, independent of the scientific merit of the study.

Adjunctive comments not previously recommended (MAJOR)

Critical Statistical Error: Significance Threshold (UNRESOLVED, PRESENT THROUGHOUT THE MANUSCRIPT)

A systematic typographical error appears repeatedly throughout the manuscript and has not been corrected in this revision. In Tables 2, 3, and 4, in the Results section, and in the Abstract, the significance threshold is consistently reported as "$p < 0.5$" instead of the correct "$p < 0.05$". The authors must conduct a thorough search of the entire manuscript including the abstract, all tables, all figure legends, and the results and discussion sections and replace every instance of "$p < 0.5$" with the correct threshold "$p < 0.05$". The authors should also verify that all reported p-values in the tables are consistent with this corrected threshold and that no conclusions change as a result.

This error must be corrected before the manuscript can be considered for publication.

3. Minor Remaining Concerns

Point 3.3 - Response Rate (STILL REQUIRES CORRECTION IN THE MANUSCRIPT)

The authors' explanation in the response letter is acceptable: collecting more responses than the minimum required sample size is a legitimate methodological decision, and including all valid responses in the analysis is appropriate. However, the manuscript itself (Methods section, p. 10) still reads: *"A total of 1085 responses were interviewed, yielding a response rate of 141.5%"*.This sentence must be corrected in the manuscript text, as a response rate exceeding 100% is a formal error that cannot appear in a published article, regardless of the explanation provided separately in the response letter.

The authors should remove the response rate figure entirely and replace it with a sentence along the following lines: *"A total of 1085 responses were collected, exceeding the minimum required sample size of 767. This was due to higher-than-expected participation in Al-Hofuf and Al-Qura. All responses were verified for completeness and validity and were included in the final analysis"*.

Point 4.1 - Comparative Prevalence Framing (MINOR)

Original concern: Labeling stigma prevalence as 'low' without methodologically comparable data.

The authors have added comparative figures from other countries but acknowledge in Point 12 that different instruments and populations were used. The revised text should include a clear caveat whenever cross-national comparisons are made, explicitly noting that differences in instruments, sampling frames, and cultural contexts limit direct comparability. A sentence to this effect should be added each time comparative data are cited in the Discussion.

Point 5.1 - Typographical Errors in the Abstract (DECLARED AS CORRECTED, STILL PRESENT)

The authors stated in their response to Point 5.1 that subject-verb disagreement had been revised throughout the manuscript. However, the abstract (p. 7) still contains two uncorrected errors. First, "The study indicate" should read "The study indicates". Second, the prevalence figure is reported as "23.1" without the percentage symbol, which should read "23.1%". Both errors remain in the revised manuscript despite the authors explicitly declaring them as corrected. The authors must carefully proofread the abstract in its entirety, as these are the first sentences a reader encounters and their presence undermines the overall quality of the manuscript.

Point 12 - Cross-National Comparisons (MINOR)

Original concern: Direct comparisons with other countries using different instruments are methodologically problematic.

The authors' response states they are "showing a trend", but the manuscript does not adequately distinguish between trend-level observations and direct comparisons. This conflation risks misleading readers. I recommend the authors revise the relevant passages to use language such as 'rates appear higher/lower than those reported in studies from X, though direct comparison is limited by differences in measurement instruments and study populations.'

4. Summary Recommendation

In its current form, the manuscript cannot be recommended for acceptance. The four major concerns identified above: instrument validation, dichotomization justification, and data availability, must be fully resolved before the manuscript can be considered further. I encourage the authors to address these points thoroughly in the next revision.

I remain willing to review a further revised version of the manuscript.

Sincerely,

Reviewer #2
