# Supplementary material for: Mental illness stigma and its impact on help-seeking behavior among residents of the Eastern Region, Saudi Arabia: A cross-sectional study
Source: PLoS One. 2026 Jun 24;21(6):e0350860. doi: 10.1371/journal.pone.0350860 (PMC13293441; doi:10.1371/journal.pone.0350860)
Supplement: Attachment — Submitted filename: Review of Manuscript_PLOS 251108.docx [file pone.0350860.s003.docx]

**Review of manuscript – PLOS**

*Mental Illness Stigma and its impact on Help-Seeking Behavior among Residents of*

*the Eastern Region, Saudi Arabia: A Cross-Sectional Study*

**General Feedback**

The content of this manuscript is relevant to the scope of PLOS. The study addresses a critical public health issue, mental illness stigma and its impact on help-seeking behavior. With 1085 participants, the study has strong statistical power and allows for meaningful subgroup analysis. The use of descriptive statistics, chi-square tests, and logistic regression provides a solid framework to explore associations and predictors.

**Feedback on abstract**

There is some inconsistency regarding gender. While the results section states gender is not significantly associated with help-seeking behavior, the conclusion suggests females are more likely to seek help. This should be clarified.

Keywords are normally in alphabetical order

**Method**

**Design and Setting**

The section is generally well-organized but could benefit from clearer separation between the study design, geographic context, and population demographics.

The proportional allocation based on population distribution enhances the generalizability of the findings within Al Ahsa.

**Instruments**

- The questionnaire is clearly structured into four parts, which helps in organizing the data collection process. However, the description of each part could benefit from more concise language. Consider summarizing the purpose of each section more efficiently to improve readability.
- The inclusion of a pilot test is a strength, demonstrating attention to clarity and feasibility. However, more detail on the sample size and demographics of the pilot group would enhance transparency.
- The reported Cronbach’s alpha of 0.83 indicates good internal consistency. It would be helpful to specify which part(s) of the instrument this reliability score applies to, e.g., stigma items only or the entire questionnaire.

**Statistical analysis**

- The mention of data cleaning in Excel is appreciated, but it would be helpful to specify what types of inconsistencies were addressed (e.g., missing values, outliers, duplicate entries). Consider clarifying whether any data were excluded during cleaning and how that may have impacted the final sample size.

**Results**

- The non-significant findings for gender, education, employment, marital status, and experience with mental illness are reported clearly. However, it would be beneficial to briefly discuss why these variables may not have shown significance, especially since prior literature often finds gender and education to be influential.

**Discussion**

- The interpretation of the 23.1% prevalence of stigma is well contextualized through international comparisons. However, the discussion could benefit from a more nuanced analysis of why stigma may be lower in your sample compared to other countries e.g., cultural factors, recent public health campaigns, or sampling methods.
- The discussion of gender differences in stigma is somewhat contradictory. You mention both consistency and inconsistency with previous studies. It would be helpful to clarify which findings your results align with and offer possible explanations for the discrepancies (e.g., cultural norms, gender roles in Saudi Arabia).

**Studylimitations**

- The limitation regarding geographic diversity is valid, especially since the study is confined to Al Ahsa. It would be helpful to specify whether this affects urban vs. rural representation or cultural variability within the region.
- While the limitations are well stated, consider explicitly mentioning how these factors may affect the generalizability of the findings to other regions or populations, especially outside Saudi Arabia.

**References**
There are inconsistencies in formatting, particularly in: Use of punctuation (e.g., inconsistent placement of periods and commas). Presentation of URLs (some are broken or contain unnecessary spacing). Journal names and volume/issue formatting (e.g., “European child & adolescent psychiatry” vs. “European Child & Adolescent Psychiatry”).Ensure adherence to the journal’s reference style guide
