# Supplementary material for: Mental illness stigma and its impact on help-seeking behavior among residents of the Eastern Region, Saudi Arabia: A cross-sectional study
Source: PLoS One. 2026 Jun 24;21(6):e0350860. doi: 10.1371/journal.pone.0350860 (PMC13293441; doi:10.1371/journal.pone.0350860)
Supplement: Attachment — Submitted filename: Response to Reviewers 17-02-2026.docx [file pone.0350860.s004.docx]

**Response to Reviewers**

| **Sr. No.** | **Comment** | **Response** | **Statement added to the body of manuscript** |
| --- | --- | --- | --- |
| **Reviewer #1:** | Thanks for this interesting paper to review, The content of this manuscript is relevant to the scope of PLOS. The study addresses a critical public health issue, mental illness stigma and its impact on help-seeking behavior. With 1085 participants, the study has strong statistical power and allows for meaningful subgroup analysis. The use of descriptive statistics, chi-square tests, and logistic regression provides a solid framework to explore associations and predictors. | We sincerely thank the reviewer for their positive and thoughtful evaluation of our manuscript. We are grateful for the reviewer’s acknowledgment of the study’s sample size, statistical power. |  |
| **Reviewer #2:** |  |  |  |
| 1 | The authors should explicitly state in the introduction what gap in the existing literature their work addresses and how it contributes beyond descriptive regional data. | We thank the reviewer for these valuable suggestions. We revise the introduction to clearly articulate the specific gap in the existing literature that our study addresses and highlight how our study can bridge the gap beyond the provision of descriptive regional data. | Based on the previous studies conducted in the Saudi context have reported the prevalence of mental illness and highlighted that only a small proportion of affected individuals receive treatment despite the availability of mental health services. However, these studies have not clearly identified the underlying reasons for low treatment uptake or the lack of social acceptance toward people with mental illness. This study contributes existing literature by examining the relationship between stigma and help-seeking behavior, offering a deeper understanding of the determinants of stigma and help seeking. |
| 2 | There is no indication that the results were published elsewhere. | The data are not published anywhere else in any form. | ----- |
| 3 | 3.1. The statistical analyses (chi-square and logistic regression) are appropriate in principle, but the operationalization of key variables is insufficiently justified. | Thanks for the both reviewers for supporting our plan of statically analysis. Respectively our justification for the key variables already discussed in our manuscript under a section of Instrument and Measurement as follows:  Key variables:   - Based on the inclusion criteria, participants aged 18 years and above were classified into categories with equal intervals of 10 years to differentiate each groups. - **The key variables**: - **Mental illness-related stigma**: The overall mental illness-related stigma was dichotomized into two categories based on the following criteria: - If score < 5 = No mental illness-related stigma. - If score ≥ 5 = there is mental illness-related stigma   Using the mean as a cutoff point was established. Where a score of less than the mean indicates no stigma and a score of more than mean indicates stigma.  **Help seeking behavior**: Likert scale questions were used, where the maximum total score was 6, with a range from 0 to 6 points. The mean used, as a cutoff point to dichotomized variables " **Help seeking behavior"** was identified based on the total range of values, where a score of: Score less than 3 indicates seeking help and a score ≤3 indicates being deterred from seeking help. | ----- |
| 3.2. | The self-developed stigma and help-seeking scales lack validation against recognized instruments (e.g., Link’s Devaluation–Discrimination Scale, or the Self-Stigma of Mental Illness Scale). | Thank you for valuable comments; We acknowledge the concern raised related validation of stigma and help-seeking scales lack validation against recognized instruments (e.g., Link’s Devaluation–Discrimination Scale, or the Self-Stigma of Mental Illness Scale).  While we admit that we did not directly validate our questionnaire against these recognized instruments , however, we take many steps to ensure the validity and reliability of our developed questionnaire which as followed:   - **Conceptual framework**: Our stigma and help-seeking questionnaire developed based on the key concepts derived from " Link’s Devaluation–Discrimination Scale", and" Self-Stigma of Mental Illness Scale", ensuring conceptual alignments with well recognized scale in the field. We aimed to capture variables related to stigma and seeking help, using these recognized scales, which are commonly used in related research. - **Content validity**: Upon formulating the questionnaire variables it's was reviewed by public health specialist to assess whether the variables adequately covered the intended domains stigma and seeking help behavior, based their comments, we modified and rephrased many variables ensuring they accurately covered the intended domains. - **Pilot test**: in addition to the revision done by public health specialist, we conduct a pilot study on a random sample from the study population. This allow us assess the reliability of the questionnaire, through Cronbach's alpha test which revealed strong internal consistency (Cronbach's alpha =0. 83), which indicating the showed reliability. | The questionnaire developed based on the key concepts derived from " Link’s Devaluation–Discrimination Scale", and" Self-Stigma of Mental Illness Scale", specifically related to the variables concerning to stigma and help-seeking in addressing psychological challenges. Some items rephrased to align with the linguistic and educational level of the study population, as well as to minimize response time. Upon formulating the questionnaire variables it's was reviewed by public health specialist to evaluate the content validity, many variables were modified or removed based on their feedback.  To assess the reliability of the questionnaire, a pilot study was conducted on a random sample from the study population. Cronbach's alpha was calculated, the results reveal the questionnaire demonstrate high validity, with a Cronbach's alpha equal (0. 83)  Link, B. G. (1987). |
| 3.3. | The reported “response rate of 141.5%” is a critical methodological error and must be corrected or clarified. | We are grateful for the reviewer's keen observation. We would like to clarify that, initial sample size was calculated using Epi Info software with an absolute precision of 5% a 95% confidence interval and a design effect 2x with resulted in a required sample size of 767. However, during data collection we received higher number of responses almost double from Al-Hofuf and respectively high response from Al-Qura too. All collected responses were subsequently checked by the researchers and were found to be complete and valid for data analysis. Therefore, we decide to include all the collected data in the final statistical analysis to reduce the error and to draw a significant conclusion. **We are thankful for Reviewer-1** for supporting our decision by mentioning that "With 1085 participants, the study has a strong statistical power and allows for meaningful subgroup analysis" | ----- |
| 3.4. | No description of multicollinearity checks or model fit diagnostics is provided for logistic regression | Thank you for valuable comments; We acknowledge the importance of assessing multicollinearity. In response to that, we assessing multicollinearity using Pearson correlations matrix variables with strong correlation (>0.6 ) were excluded from the model. | ----- |
| 3.5 | Dichotomization thresholds (e.g., “<5 indicates no stigma”) are arbitrary and require theoretical or empirical justification | We thank the reviewer for the insightful comment regarding the dichotomization thresholds. In this study, we used the mean score as the cutoff point. A score below the mean indicates no mental illness related stigma, whereas a score equal to or above the mean indicates the presence of mental illness related stigma. Accordingly:   - Mean score < 5 indicate no mental-illness–related stigma - Mean score ≥ 5 indicate no Presence of mental-illness–related stigma   This approach to dichotomization using the mean as a cutoff point has been reported in previous studies, conducted by Lapham et al., 2022 and Hadera et al., 2019)   - Lapham, J., & Martinson, M. L. (2022). The intersection of welfare stigma, state contexts and health among mothers receiving public assistance benefits. *SSM-population health*, *18*, 101117. - Hadera, E., Salelew, E., Girma, E., Dehning, S., Adorjan, K., & Tesfaye, M. (2019). Magnitude and associated factors of perceived stigma among adults with mental illness in Ethiopia. *Psychiatry journal*, *2019*(1), 8427561. | ----- |
| 4 / 4.1. | Conclusions are presented in an appropriate fashion and are supported by the data. Conclusions are generally consistent with the data, but they are overstated . For instance, labeling the prevalence of stigma as “low” is not justified without comparative, methodologically similar data | In the discussion section, we have already compared our findings on the prevalence of stigma with those from developed and developing countries, and we have made the necessary corrections in the conclusion section based on the reviewer’s comments. As eg :  " The prevalence was found to be lower in developed nations like the United States ^(53)^ compared to developing countries such as Nigeria (41.5%) ^(54)^ Qatar (41.8%) ^(55)^ and China (49.5%), ^(56)^. " | ----- |
| 4.2. | The claim that “young adults and females are more likely to seek help” should be discussed in a socio-cultural context rather than simply restating numerical findings. It requires significant revision for interpretative depth and caution. | Thanks to reviewer for their constructive feedback. We acknowledged the need of a deeper, socio-culture interpretation of the finding that "young adults and females are more likely to seek help".  We elaborated this issue in detail within our **Discussion** section supported by adding new reference for the similar context. | Generally this reflect the generational shift young adults, are increasingly inclined to seek assistance, The younger generation has become more open to mental health discussions as a result of both local and global awareness campaigns and social media influence, which contributed in their healthy literacy and enhanced their understanding of mental illnesses. Among young adults, females have increasingly engaged with mental health services due to the societal changes, greater awareness and a greater tendency toward emotionally expressive to seek support when facing challenges.   However, males, particularly older individuals, may face stigma and cultural barriers that discourage seeking help (Mahsoon etal 2020) ^(70)^ . The culture expectation of masculinity also discourage emotional expression and help seeking behaviors, as males are expected to handle their problem independently and without external assistance. This may scaling up stigma around mental illnesses and further discouraging individuals from seeking help from the appropriate sources (Nagai, S 2023) ^(71).^  Taken together, these findings suggest that efforts to reduce mental-illness stigma and promote help-seeking should consider age- and gender-specific sociocultural factors. Targeted interventions for younger adults may leverage their openness and access to information, while strategies aimed  fir men and residents of certain regions should address cultural barriers and societal expectations that inhibit help-seeking within Saudi community .    Mahsoon, A., Sharif, L., Banakhar, M., Alasmee, N., Almowallad, E., Jabali, R., ... & Assur, S. (2020). Parental support, beliefs about mental illness, and mental help-seeking among young adults in Saudi Arabia. International journal of environmental research and public health, 17(15), 5615.  Nagai, S., Kimura, M., Honda, M., Iida, T., & Mizuno, H. (2023). Gender Differences in Help‐Seeking: A Meta‐analysis of Japanese Studies, Including Unreported Data 1. Japanese Psychological Research. |
| 5.1. | Subject–verb disagreement (“The study indicate…” → “The study indicates”). | Thank you for the comment. The sentences has been revised using correct verbs. | ----- |
| 5.2. | Inconsistent terminology (“mental illness stigma”, “mental health stigma”). | Thanks for the reviewers keen observation correction are done throughout the manuscript as suggested. | ----- |
| 5.3. | Redundant phrasing and repetition throughout. Requires thorough language editing by a native or professional scientific editor. | Done | ----- |
| 6./ 6.1. | Ethical approval and informed consent are appropriately stated (Ethical criteria met). | Yes , Thanks | ----- |
| 6.2. | However, the Data Availability section is inconsistent: the form declares “data are fully available”, while the manuscript says “available upon reasonable request”. PLOS ONE requires unrestricted data availability. This inconsistency must be resolved | We are following the PLOS ONE criteria, and we are submitting our data as a **supplementary file** attaching along with our revision. | ----- |
| 7/ 7.1. | The paper lacks adherence to established reporting guidelines for cross-sectional studies (e.g., STROBE). | We thank the reviewer for their valuable comment. In response, we carefully reviewed the manuscript to ensure alignment with the STROBE reporting guidelines for cross-sectional studies. We confirms that our manuscript already following the relevant STROBE guidelines reported as follows :  **Title**:   - The study design is now clearly stated in the title " Mental Illness Stigma and its impact on Help-Seeking Behavior among Residents of the Eastern Region, Saudi Arabia: A Cross-Sectional Study"   **Abstract**:   - The study design was clearly stated in the abstract (Methods: first line).   **Introduction**:   - We strengthened the rationale and clearly stated the study objectives.   **Methods** :   - The study design, study population, study setting, participant recruitment, and inclusion/exclusion criteria have been clarified. - Variable definitions have been standardized and are now consistent across all tables. - Data sources, measurement procedures, and potential sources of bias are explicitly described. - Sample size justification has been added. - Statistical methods have been expanded, with clearer explanations of multivariable models and effect estimates.   **Results**:   - Participant flow is now clearly reported - Deceptive and analytical are presented with corresponding effect sizes and 95% confidence intervals.   **Discussion**:   - The main findings are summarized in relation to study objectives and explained clearly, while the study limitations are fully addressed. - With regard, the Interpretation the study results compared with others studies. since we are surveying large sample size and the participants recruited through random methods the results can be generalized to other settings .   **Funding**:   - A clear statement on funding sources and roles has been included.   Finally, we appreciate the reviewer’s support, as these revisions have significantly improved the overall quality of the manuscript.  *Note: We have also reviewed the full STROBE checklist to ensure comprehensive reporting and confirm that the revised manuscript now adheres to the recommended standards. | ----- |
| 7.2. | Tables are clear but lack clear variable definitions and labeling consistency. | We have revised the variables presented in the tables 1,3 and 4. Especially in Table 5, we noticed that some data were swapped between employment and marital status during our original submission. Although this error did not affect our findings, we have now corrected it. | ----- |
| 7.3. | The instrument development process should be described in detail, including item examples and scoring rationale. | The instrument is developed by authors based on the literature review, expert consultation, focus group discussions also guided by the general concept of the stigma scale such as  Link’s Devaluation–Discrimination Scale, or the Self-Stigma of Mental Illness Scale. In order to be consistent with the values, culture and other social factors.  The **Methods section**- **under Instrument and Measurement** has include a clearer description of the instrument development process, including item generation, representative examples, and the rationale for scoring. | Added relevant information in **Methods section**- **under Instrument and Measurement** section. |
| 8/ 8.1. | Major Comments 1. Conceptual Framing  The introduction should include a stronger theoretical grounding in stigma research (e.g., Corrigan & Watson’s framework; Link & Phelan’s model). The current framing is descriptive rather than analytical | We thank you for this valuable comment. We agree that a stronger theoretical grounding enhances the analytical depth of the topic background. In response we have revised the topic background to explicitly incorporate established stigma framework including the Corrigan & Watson’s framework; Link & Phelan’s model (labeling, stereotyping status loss and discrimination). These frameworks are now used to guide the conceptual framing of mental illness stigma and to analytically link stigma to help seeking behavior rather than description.  Based on reviewer suggestions we updated our **Introduction** section with addition of supporting references accordingly (Corrigan & Watson’s framework; Link & Phelan’s model…etc). . | Existing literature has shown that mental illness is associated with stigma which refers to negative attitudes, beliefs, stereotypes held by the public towards individuals with mental illness, leading to societal prejudice and discrimination against them (Al-Hashemi et al., 2025), (Link et al., 2001). Psychological frameworks describe stigma as encompassing stereotypes, labeling, isolation, status loss, prejudice, and discrimination (Corrigan et al., 2011). Sigma can appear in various forms including public stigma and self- stigma, structural stigma and perceived stigma all of which influences the behaviors and willingness of individuals and families affected by mental illness to seek help (Link et al., 2001).  Corrigan et al., reported that common stereotypes associated with individual with serious mental illness includes dangerousness, blame, and social incompetence (Corrigan, 2005). Moreover, some previous studies have sought to conceptualize stereotypes that contribute self-stigma, which significantly influence help seeking behaviors, for example Ritsher et al and others developed a five factor scale to assess self-stigma which includes alienation, stereotype endorsement, discrimination experience, social withdrawal and stigma resistance. This model was guided by different scale (Link, 1987, Corrigan et al., 2006; Watson et al., 2007) used to examine whether individuals with mental illness are recognize the stereotypes related to mental illness. This conceptualization of stigma as progressive experience is critical in understanding how stigma can influence help seeking behavior the process begin with individuals awareness about societal stereotypes surrounding metal illness followed by agreement, where internalization these stereotypes as a facts it will lead to the application of these negative believe to oneself, resulting in server consequences such as reduce self-esteem and hesitant to seek help (Corrigan et al., 2006) |
| 9/ 9.1. | Instrument Validation  Provide detailed information on how the stigma and help-seeking scales were developed, tested, and validated. | The Methods section has been revised to include a clearer description of the instrument development, testing and validation process (**Methods section**- **under Instrument and Measurement)**  The instrument used in this study was a researcher-developed questionnaire, to ensure clarity, relevance, and cultural appropriateness. Additionally, the questionnaire reviewed by public health specialist to evaluate the content validity.  Moreover, reliability was assessed using Cronbach’s alpha and questionnaire demonstrated good internal consistency, with a Cronbach’s alpha test result of (0.83). | ----- |
| 9.2. | If they are novel, include factor analysis and reliability indices beyond Cronbach’s alpha. | We thank the reviewer for this valuable comment. Our stigma and help-seeking questionnaire were self-developed after rigorous literature review, expert consultation, focus group discussions also guided by the general concept of the stigma scale (" Link’s Devaluation–Discrimination Scale" ^(13)^, and" Self-Stigma of Mental Illness Scale" ^(51)^,), to address specific aspects of mental illness stigma in Saudi context. So it's not a novel scale, but a self-developed questionnaire to estimate the prevalence of mental illness stigma and exploring the association of the stigma with help-seeking behavior. | " Link’s Devaluation–Discrimination Scale" ^(13)^, and" Self-Stigma of Mental Illness Scale" ^(51)^. |
| 10/ 10.1. | Statistical Analysis Explain the rationale for the dichotomization of stigma and help-seeking scores. Consider reanalyzing the data using continuous or ordinal scales to preserve statistical power. | We thank the reviewer for the valuable comments. But, we would like to clarify that the primary objective of the study is to determine the level of stigma in the Saudi community and the factors associated with it. So,   - The rationale behind using the dichotomization to estimate the prevalence of stigma, the mean score was used as the cutoff to identify individuals with stigma toward mental illnesses. - Since the outcome variable was dichotomized, we selected the appropriate statistical tests (**A multivariate binary logistic regression analysis was used**) that can preserve statistical power accordingly. | ----- |
| 11. | Interpretation of Results: The discussion section should distinguish between association and causation. Statements implying that stigma “leads to” help-seeking avoidance should be rephrased to reflect a correlational design. | We thank the reviewer for this perceptive comment.   - We have revised the discussion section to clarify that we did not use statements that reflect the causation such as "lead to". - Although in introduction section, we cited some studies; reporting that stigma lead to multiple negative factors such as disability, substance abuse, homelessness …etc. | No Change:  "Previous studies suggest that levels of stigma are the main stumbling block preventing patients from seeking mental health consultation and creating a gap between community, service, and treatment (30) and lead to different negative outcomes such as distress, low self-esteem, self-efficacy, treatment delay, treatment drop-out, exacerbating maladaptive behaviors, and reduced help-seeking behaviors. (34)" |
| 12 | Comparative Context:  Avoid direct comparisons with prevalence rates from other countries unless similar instruments and populations were used. Instead, emphasize contextual explanations (e.g., cultural norms, healthcare infrastructure). | Thank the reviewer for his judgmental comments: here I would like to show a trend and trying to rationalize our findings scientifically. We are not comparing the data as acquired data through different methodology. | ----- |
| 13 | Data Transparency:  Make all de-identified data and the full questionnaire publicly available (e.g., via a repository link). | We thank the reviewer for emphasizing data transparency. In accordance with PLOS ONE policies, the full questionnaire can be made available upon request. Additionally, if required, we are willing to submit the material as a supplementary file during the final revision. | ----- |
| 14 | Language and Readability:  The manuscript needs comprehensive professional English editing to meet publication standards. | We confirm that the manuscript has been fully edited by a linguistic to ensure clarity and proper use of English grammar. | ----- |
|  | Minor Comments |  |  |
| 15 | Check consistency of numerical data between abstract, results, and tables. | We are thankful for reviewer for highlighting this point. We have carefully reviewed the manuscript and ensured that all numerical data are consistent across the abstract, result section and revised the tables wherever required. |  |
| 16 | Correct typographical and formatting inconsistencies (e.g., decimal points, percentage spacing). | DONE | ----- |
| 17 | Ensure all references follow PLOS ONE style (complete DOI, correct capitalization). | Thanks for this suggestion from reviewers, We ensure that we will update all the references according to the PLOS ONE style during the proofreading stage.  In addition, we have added new supporting references to strengthen the manuscript | ----- |
| 18 | Revise phrases like “the study indicate that” and “vice versa” for clarity and conciseness. | Revised as suggested by reviewer | ----- |
| 19 | Replace “low” or “high” with quantitative descriptors where possible (e.g., “23.1%, lower than in prior regional studies”). | Thanks for this comment we revised it accordingly. | ----- |
| 20 | Verify that the ethical approval number matches the institutional record. | The ethical approval number has been verified and matches the institutional record (KFU-REC-2024-OCT-ETHICS2653). | ----- |
