# Supplementary material for: Mental illness stigma and its impact on help-seeking behavior among residents of the Eastern Region, Saudi Arabia: A cross-sectional study
Source: PLoS One. 2026 Jun 24;21(6):e0350860. doi: 10.1371/journal.pone.0350860 (PMC13293441; doi:10.1371/journal.pone.0350860)
Supplement: Attachment — Submitted filename: Review 260226 PLOS one.docx [file pone.0350860.s005.docx]

Review PLOS

Mental Illness Stigma and its impact on Help-Seeking Behavior among Residents of the Eastern Region, Saudi Arabia: A Cross-Sectional Study

Dear Authors,

Thank you for the opportunity to review your manuscript again. The topic is both timely and relevant, and your work contributes meaningfully to the understanding of Mental Illness Stigma and its impact on Help-Seeking Behavior a thorough evaluation, I offer the following comments and suggestions to enhance the clarity, consistency, and overall quality of the paper.

**Comment 1** – Keywords

The keywords should be arranged in alphabetical order to comply with standard manuscript formatting.

**Comment 2** – Methods

- Please ensure adherence to the STROBE checklist for observational studies to improve transparency and completeness.
- You did not state how participants were selected (convenience sampling? random sampling?)
- No mention whether language versions were used or validated equally

**Comment 4** – Results

The result is of interest check that titles are on correct line,

**Comment 5** – Discussion

The discussion may be considered to put in sections, what you want to discuss, right now there is a mix. The beginning is more like a background section.
What did you find? What does this mean for practice or policy? Your findings (e.g., stigma levels, help‑seeking barriers, rural–urban differences, gender perspectives, multivariate associations) are barely contextualized. There is a bit too much unrelated epidemiological data.

**Comment 6** – Limitations

The manuscript clearly acknowledges several limitations, and I appreciate that these are presented transparently. I would, however, encourage you to further refine and elaborate on these points to strengthen the methodological rigor of the discussion. The reliance on self‑reported data for assessing prevalence and help‑seeking behavior is indeed a limitation, as such measures are prone to recall bias and potential social desirability bias. How could this have influenced the direction or magnitude of your findings.

Although you correctly note that the cross‑sectional design precludes causal inference, you may strengthen this section by briefly describing how temporal or longitudinal approaches in future studies could help clarify the directionality of relationships between stigma, influencing factors, and help‑seeking behaviors.

**Comment 7** – Conclusion

Your study presents valuable insights into mental illness–related stigma and its relationship with help‑seeking behavior. The comparison with neighboring countries, the identification of key sociocultural factors influencing stigma, and the multivariate findings regarding age and rural residence contribute meaningfully to the regional understanding of mental health attitudes

**Comment 8. References and formatting**

It is difficult to thoroughly review the manuscript in its current form due to the absence of line numbers and insufficient paragraph structure. Adding line numbers and improving the paragraph division would greatly facilitate a more precise and constructive review and make it easier for the reader.

References vary, sometimes tendency of APA 7 and sometimes tendency of Vancouver, this need to be checked out. In text another reference system.
