# Supplementary material for: Mental illness stigma and its impact on help-seeking behavior among residents of the Eastern Region, Saudi Arabia: A cross-sectional study
Source: PLoS One. 2026 Jun 24;21(6):e0350860. doi: 10.1371/journal.pone.0350860 (PMC13293441; doi:10.1371/journal.pone.0350860)
Supplement: Attachment — Submitted filename: Response_R2_04_04_26.docx [file pone.0350860.s007.docx]

**Response Vol-2**

| **Sr. No.** | **Comment** | **Response** | **Statement added to the body of manuscript** |
| --- | --- | --- | --- |
| **Reviewer #1:** |  |  |  |
| **Comment 1: Keywords** | The keywords should be arranged in alphabetical order to comply with standard manuscript formatting. | Updated as suggested by reviewer. | Behaviors, Mental illness, Prevalence, Saudi Arabia, Seeking, Stigma. |
| **Comment 2** – Methods | 2.1  Please ensure adherence to the STROBE checklist for observational studies to improve transparency and completeness. | We thank the reviewer for his valuable comment. We carefully reviewed the manuscript to ensure alignment with the STROBE reporting guidelines for cross-sectional studies. We confirm that our manuscript already following the relevant STROBE guidelines. (We explained it in details during our **1^st^ Response To Reviewers** : section :Reviewer-2 : comment section 7.1) |  |
|  | 2.2  You did not state how participants were selected (convenience sampling? random sampling?) | We acknowledged reviewer comment: We explained it page 4: Methods: Sample techniques: line 32. |  |
|  | 2.3  No mention whether language versions were used or validated equally. | The questioner originally developed in English and later translated by a native speaker for Arabic version. Page 5 : line 14-15. |  |
| **Comment 4** – Results | 4.1 The result is of interest check that titles are on correct line. | We thanks the reviewer for his observation, we have carefully checked the manuscript and ensured that all the titles are correctly aligned with appropriate section. |  |
| **Comment 5** – Discussion | 5.1 The discussion may be considered to put in sections, what you want to discuss, right now there is a mix. The beginning is more like a background section. What did you find? What does this mean for practice or policy? Your findings (e.g., stigma levels, help‑seeking barriers, rural–urban differences, gender perspectives, multivariate associations) are barely contextualized. There is a bit too much unrelated epidemiological data. | Thanks for the reviewer. We updated our discussion in different sections based on the given suggestion of reviewer. As   - Prevalence of stigma- related mental illnesses - Gender-based differences in prevalence of mental illness stigma - Factors Associated with Mental Illness Stigma - Help-seeking behavior and associated factors   Page 11 -13. |  |
| **Comment 6** – Limitations | 6.1:  The manuscript clearly acknowledges several limitations, and I appreciate that these are presented transparently. I would, however, encourage you to further refine and elaborate on these points to strengthen the methodological rigor of the discussion. The reliance on self‑reported data for assessing prevalence and help‑seeking behavior is indeed a limitation, as such measures are prone to recall bias and potential social desirability bias. How could this have influenced the direction or magnitude of your findings.  Although you correctly note that the cross‑sectional design precludes causal inference, you may strengthen this section by briefly describing how temporal or longitudinal approaches in future studies could help clarify the directionality of relationships between stigma, influencing factors, and help‑seeking behaviors. | We updated our limitation section as suggested and added in the body of manuscript. From line 30 to 36. | There are many limitations that need to be taken into consideration. First: the assessment of prevalence and help-seeking behavior depends on participants’ self-reports, which may be subject to recall bias and social desirability bias, which may limit the accuracy of the assessment. To mitigate these potential biases, we emphasized anonymous and confidential data collection and structured questions to reduce judgmental framing. Second: content validity: In absence of conducting factor analysis, content validity was assessed based on rigorous literature review, expert consultation, focus group discussions also guided by the general concept of the stigma scale (" Link’s Devaluation–Discrimination Scale" (13), and" Self-Stigma of Mental Illness Scale (51), ", which may affect the generalizability of the results. Third: this research faces limitations regarding the diversity of the geographic regions represented in the sample. Fourth, the cross-sectional study design may preclude any causal relationship from being established between influencing factors and formal help-seeking. Future studies should consider these limitations, thus increasing the research results' applicability and utilization. |
| **Comment 7** – Conclusion | 7.1 Your study presents valuable insights into mental illness–related stigma and its relationship with help‑seeking behavior. The comparison with neighboring countries, the identification of key sociocultural factors influencing stigma, and the multivariate findings regarding age and rural residence contribute meaningfully to the regional understanding of mental health attitudes. | We sincerely thank the reviewer for this encouraging feedback. We are pleased that the regional comparisons, identification of sociocultural factors, and multivariate findings were found to add meaningful insights in the manuscript. |  |
| **Comment 8. References and formatting:** | 8.1 It is difficult to thoroughly review the manuscript in its current form due to the absence of line numbers and insufficient paragraph structure. Adding line numbers and improving the paragraph division would greatly facilitate a more precise and constructive review and make it easier for the reader. | We thank the reviewer for this suggestion.  We have now added line numbers to the revised manuscript. Also we tried to improved paragraph structure throughout the text to make the manuscript easier to read and review. |  |
|  | 8.2 References vary, sometimes tendency of APA 7 and sometimes tendency of Vancouver, this need to be checked out. In text another reference system. | Thanks for the keen observation of the reviewer, Yes we ensure that , we will update all the reference based on the journals requirements, Once this manuscript is consider for further processing. |  |
| **Reviewer #2:** |  |  |  |
| **Comment 1:** | **Considered Satisfactorily Addressed**  The following points from the first round have been addressed to a satisfactory degree and require no further revision:  • 3.4 (multicollinearity check), 5.1–5.3 (language corrections), 6.1 (ethical approval), 7.1 (STROBE alignment), 7.2 (table corrections), 8.1 (theoretical grounding), 11 (causation vs. correlation language), 15–20 (minor corrections). | We sincerely thank the reviewer for their constructive feedback. |  |
| **Comment 2: (Point 3.2 and 9.1 and 9.2 -)** | **Major Outstanding Concerns**  **Point 3.2 and 9.1 and 9.2 - Instrument Validation (MAJOR, UNRESOLVED)**  *Original concern: The self-developed stigma and help-seeking scales lack validation against recognized instruments. Factor analysis and additional reliability indices were requested.*  The authors’ response acknowledges the absence of formal validation but argues that conceptual alignment with Link's Devaluation–Discrimination Scale and the Self-Stigma of Mental Illness Scale is sufficient. This is not acceptable for a PLOS ONE submission. Conceptual alignment during item generation is a preliminary step, not a substitute for psychometric validation.  Specifically:  (a) The claim that the questionnaire is “not a novel scale” and therefore does not require factor analysis is incorrect. Any self-developed instrument, regardless of its conceptual origins, requires structural validation particularly when used to produce prevalence estimates that form the primary outcome of the study.  (b) Cronbach’s alpha (0.83) measures internal consistency but provides no evidence of construct validity, dimensionality, or criterion-related validity.  (c) The revised manuscript should either: (i) report an exploratory or confirmatory factor analysis to establish the dimensional structure of the instrument; or (ii) include an explicit acknowledgment in the Limitations section that the absence of formal psychometric validation is a significant constraint on the generalizability and comparability of findings. This limitation must be discussed substantively, not simply mentioned in passing. | Based on our first response to reviewer section 3.2, 9.1, 9.2, we clarify that our questionnaire was developed through a rigorous literature review, expert consultation, and based on the key concepts derived from " Link’s Devaluation–Discrimination Scale", and" Self-Stigma of Mental Illness Scale", specifically related to the variables concerning to stigma and help-seeking behavior. Additionally, we already added those details in our previous revision of manuscript page 5: Instrument and measurements section: Line 28 - 33.On the other hand, the primary aim of this study was to estimate the prevalence of mental illness stigma and explore its association with help-seeking behavior, the questionnaire provides a valid, reliable, and contextually appropriate instrument for these objectives.  The approach used by us to ensure the content validity of the questionnaire mentioned above, has been employed in many studies published in reputed Public Health peer-reviewed journals, such as American Journal Of Mental Health , BMC Public Health and PLOS One.  <https://doi.org/10.1177/1557988318773656>  <https://link.springer.com/article/10.1186/s12889-019-7915-6>  <https://doi.org/10.1371/journal.pone.0211033> Given this, we recognize the importance of conducting a factor analysis as an additional procedure to confirm content validity. However, in this study, it was not performed because the sample collected in the pilot study was not large enough for a factor analysis test. This limitation was acknowledged in the study limitations section.  We respectfully submit that the current approach is sufficient to support the main outcomes of our study, and we believe that the manuscript meets the standards for publication in PLOS ONE. | Limitation : content validity: In absence of conducting factor analysis, content validity was assessed based on rigorous literature review, expert consultation, focus group discussions also guided by the general concept of the stigma scale (" Link’s Devaluation–Discrimination Scale" ^(13)^, and" Self-Stigma of Mental Illness Scale ^(51)^, ", which may affect the generalizability of the results. |
| **Comment 2.1: (Point 3.5 and 10.1_A)** | **-Dichotomization Thresholds (MAJOR, PARTIALLY RESOLVED)**  *Original concern: Dichotomization thresholds are arbitrary. Reanalysis using continuous or ordinal scales was recommended.*  The authors justify using the mean as a cutoff by citing two previous studies (Lapham et al., 2022; Hadera et al., 2019). This justification remains weak. Neither reference appears to use a comparable instrument or study design, and citing precedent for a practice does not resolve its methodological limitations. | We agree with the reviewer point of view, However we wanted to mention that, selecting a mean to dichotomize or categorize stigma level is a common methodological challenge. It remains a valuable tool for simplifying complex data for broader communication, because it represented the sample’s central tendency in a field of lacking of practical cutoffs.   However, to support our study we conducted a literature review and found similar practices and standard studies published in renowned journals such as Plos One, BMC Public health.  Although these studies may use different instruments, the methodological backbone remains consistent with our own. For instance, Wahyuhadi et al. utilized a cross-sectional design and categorized stigma levels using the mean score as a cutoff point. Similarly, Chang et al. employed a cross-sectional design with a self-stigma scale, classifying the level of stigma using the same mean score as a cut off point. We believe this approach provides a consistent structure for interpreting our findings within the existing literature.   Reference :   - Wahyuhadi J, Efendi F, Al Farabi MJ,   Harymawan I, Ariana AD, Arifin H, et al. (2022) Association of stigma with mental health and quality of life among Indonesian COVID-19 survivors. PLoS ONE 17(2): e0264218. <https://doi.org/10.1371/journal.pone.0264218>   - Chang K-C, Lin C-Y, Chang C-C, Ting S-Y, Cheng C-M, Wang J-D (2019) Psychological distress mediated the effects of self-stigma on quality of life in opioid-dependent individuals: Across-sectional study. PLoS ONE 14(2): e0211033. <https://doi.org/10.1371/journal.pone.0211033> - Datiko, D. G., Jerene, D., & Suarez, P. (2020). Stigma matters in ending tuberculosis: Nationwide survey of stigma in Ethiopia. *BMC public health*, *20*(1), 190. <https://doi.org/10.1186/s12889-019-7915-6> |  |
| **Comment 3: (Point 3.5 and 10.1_B)** | Furthermore, note that the response document itself contains an internal contradiction: under Point 3.5, it states both 'Mean score ≥ 5 indicates no mental illness-related stigma' AND 'indicates presence of mental illness-related stigma', these are mutually exclusive and suggest the revision was not reviewed carefully. | We appreciate the reviewer’s careful reading, which helped us correct this mistake. We apologize for the oversight. Yes it was a typo error in our last response to reviewer file only. Now it has been corrected in both the response document and the manuscript to ensure consistency, as follows.   In this study, we used the mean score as the cutoff point. A score below the mean indicates no mental illness related stigma, whereas a score equal to or above the mean indicates the presence of mental illness related stigma. Accordingly:   - Mean score < 5 indicate no mental-illness–related stigma - Mean score ≥ 5 indicate presence of mental-illness–related stigma |  |
| **Comment 3: (Point 3.5 and 10.1_C)** | Furthermore, the Methods section (p. 12) contains another logical contradiction in the definition of the help-seeking cutoff that was not present in the response letter but appears in the revised manuscript: *“a score of less than 3 indicates seeking help and a score <3 indicates being deterred from seeking help”.* Both conditions use the same threshold (<3) to define two mutually exclusive outcomes, which is logically impossible and renders the operationalization of the outcome variable unintelligible. | Thanks for the reviewer.  Corrected as suggested in the manuscript . > 3.  Page 6 , line 3 |  |
| **Comment 3: (Point 3.5 and 10.1_D)** | The authors should at minimum: (i) correct the internal contradictions noted above in both the response document and the manuscript; | Done: Corrected in the manuscript body and response to reviewer’s. |  |
|  | (ii) provide a more substantive theoretical or empirical rationale for the specific cutoff of 5 (not simply that the mean was used);  (iii) acknowledge in the Limitations section that dichotomization leads to loss of information and may inflate or deflate prevalence estimates depending on the distribution of scores. | We thank the reviewer for requesting a statistical rationale for using the mean score as a cutoff for dichotomization. The mean represents the central tendency of the sample and provides a data-driven threshold to categorize participants responses, it is also a common approach in health-related research (as mentioned in Response to reviewer-2 : points 3.5 along with references).  Specifically, the mean was used as the cutoff because:   - It represents the central tendency of the data, - It provides an objective threshold to distinguish higher versus lower levels of the variable; - It is sample-specific, and ensuring the cutoff reflects the distribution of responses rather than an arbitrary or externally imposed value. |  |
| **Comment 4: (Point 6.2 and 13)** | **Data Availability (UNRESOLVED, POLICY VIOLATION)**  This issue was raised in the first round of review and remains unresolved. The submission form contains a direct contradiction: the authors selected “Yes - all data are fully available without restriction” but then wrote in the text box below: *“supplementary data can be provided on request”.* These two statements are mutually exclusive. Additionally, the Data Availability statement in the manuscript (p. 13) states that data are "available as supplementary file" without providing any repository link, DOI, or accession number.  PLOS ONE’s data availability policy is unambiguous: data must be fully and unconditionally available at the time of publication, without requiring readers to contact the authors. Stating that data are available "on request" or "as a supplementary file" without a retrievable location does not meet this requirement.  To comply, the authors must take one of the following actions before resubmission:   1. Deposit the full de-identified dataset in a recognized public repository (e.g., Figshare, Zenodo, OSF, or Dryad), obtain a DOI, and include it in the Data Availability Statement; or 2. Upload the dataset as a formal supplementary file through the journal submission system, ensuring it is publicly accessible upon publication - not upon request.   The Data Availability Statement in the manuscript must then be updated to reflect exactly where and how the data can be accessed, with a direct link or DOI. Failure to resolve this point may result in rejection on editorial policy grounds, independent of the scientific merit of the study. | We thank the reviewer for noting the issue regarding data availability.  To comply fully with PLOS ONE’s data policy, we have deposited the complete de-identified dataset in Figshare,(<https://doi.org/10.6084/m9.figshare.31811980.v2>).  Which is publicly accessible at: Figshare and a supplementary file through the journal submission system.  The Data Availability statement in the manuscript has been updated at page 14 . With this update, the dataset is fully and unconditionally accessible to all readers without requiring contact with the authors. We confirm that this point has now been resolved in accordance with PLOS ONE policy. | The datasets used for this study are publicly available in Figshare at <https://doi.org/10.6084/m9.figshare.31811980.v2>” as well as supplementary file through the journal submission system. |
| **Comment 5:** | **Adjunctive comments not previously recommended (MAJOR)**  **Critical Statistical Error: Significance Threshold (UNRESOLVED, PRESENT THROUGHOUT THE MANUSCRIPT)**  A systematic typographical error appears repeatedly throughout the manuscript and has not been corrected in this revision. In Tables 2, 3, and 4, in the Results section, and in the Abstract, the significance threshold is consistently reported as “p < 0.5” instead of the correct “p < 0.05”. The authors must conduct a thorough search of the entire manuscript including the abstract, all tables, all figure legends, and the results and discussion sections and replace every instance of “p < 0.5” with the correct threshold “p < 0.05”. The authors should also verify that all reported p-values in the tables are consistent with this corrected threshold and that no conclusions change as a result.  This error must be corrected before the manuscript can be considered for publication. | We thank to reviewer for carefully identifying this typo error. We have review it thoroughly in the entire manuscript, including the abstract , result section , discussion , Table 2,3, and 4 along with all figure ligand. All instances of “p < 0.5” have been corrected to the accurate threshold of “p < 0.05.”  Additionally we have verified that all reported p-values in the manuscript are consistent with this correct threshold  (p < 0.05) |  |
| **Comment 6: (Minor Remaining Concerns**  **Point 3.3)** | **Response Rate (STILL REQUIRES CORRECTION IN THE MANUSCRIPT)**  The authors' explanation in the response letter is acceptable: collecting more responses than the minimum required sample size is a legitimate methodological decision, and including all valid responses in the analysis is appropriate. However, the manuscript itself (Methods section, p. 10) still reads: *“A total of 1085 responses were interviewed, yielding a response rate of 141.5%”.*This sentence must be corrected in the manuscript text, as a response rate exceeding 100% is a formal error that cannot appear in a published article, regardless of the explanation provided separately in the response letter.  The authors should remove the response rate figure entirely and replace it with a sentence along the following lines: *“A total of 1085 responses were collected, exceeding the minimum required sample size of 767. This was due to higher-than-expected participation in Al-Hofuf and Al-Qura. All responses were verified for completeness and validity and were included in the final analysis”.* | We thanks the reviewer and updated as suggested. Page 5 : line 2-5 . | A total of 1085 responses were collected, exceeding the minimum required sample size of 767. This higher number was due to the higher-than-expected participation in Al hofuf and Al Qura. All responses were verified for completeness and validity and were included in the final analysis. |
| **Comment 7: (Point 4.1)** | **Comparative Prevalence Framing (MINOR)**  *Original concern: Labeling stigma prevalence as 'low' without methodologically comparable data.*  The authors have added comparative figures from other countries but acknowledge in Point 12 that different instruments and populations were used. The revised text should include a clear caveat whenever cross-national comparisons are made, explicitly noting that differences in instruments, sampling frames, and cultural contexts limit direct comparability. A sentence to this effect should be added each time comparative data are cited in the Discussion. | Thanks for reviewer, theses intelligent suggestions helped a lot to improve the quality of manuscript. A sentence to this effect is added in the manuscript. Page 11: Line 23- 24 and 40 -41. | (These comparisons should be interpreted cautiously due to the differences in instruments, sampling and cultural context). |
| **Comment 8: (Point 5.1)** | **Typographical Errors in the Abstract (DECLARED AS CORRECTED, STILL PRESENT)**  The authors stated in their response to Point 5.1 that subject-verb disagreement had been revised throughout the manuscript. However, the abstract (p. 7) still contains two uncorrected errors. First, “The study indicate” should read “The study indicates”. Second, the prevalence figure is reported as “23.1” without the percentage symbol, which should read “23.1%”. Both errors remain in the revised manuscript despite the authors explicitly declaring them as corrected. The authors must carefully proofread the abstract in its entirety, as these are the first sentences a reader encounters and their presence undermines the overall quality of the manuscript. | We are thankful for reviewer for this keen observation, I think we missed it in the final version while saving our file. But now we corrected it as suggested. Page 1: Line 33. | “The study indicates” and 23.1% |
| **Comment 8: (Point 12)** | **Cross-National Comparisons (MINOR)**  *Original concern: Direct comparisons with other countries using different instruments are methodologically problematic.*  The authors’ response states they are “showing a trend”, but the manuscript does not adequately distinguish between trend-level observations and direct comparisons. This conflation risks misleading readers. I recommend the authors revise the relevant passages to use language such as 'rates appear higher/lower than those reported in studies from X, though direct comparison is limited by differences in measurement instruments and study populations. | We agree with the reviewer's meticulous assessment. It appears we were not able to deliver our statement clearly in our earlier revision of Point 12.  We aimed to highlight this trend by stating in the manuscript that prevalence was lower in developed nations. We intentionally included specific figures for developing countries such as Nigeria (41.5%) (54), Qatar (41.8%) (55), and China (49.5%) to provide immediate clarity for the reader. Our goal was to present these details directly in the text to prevent the reader from needing to cross-reference the citations for basic statistical details.  Page 11: line 12. |  |
