# Supplementary material for: Mental illness stigma and its impact on help-seeking behavior among residents of the Eastern Region, Saudi Arabia: A cross-sectional study
Source: PLoS One. 2026 Jun 24;21(6):e0350860. doi: 10.1371/journal.pone.0350860 (PMC13293441; doi:10.1371/journal.pone.0350860)
Supplement: Attachment — Submitted filename: Response to reviewers 3rd.docx [file pone.0350860.s008.docx]

**Response to Editorial team and reviewer’s**

| **Sr. No.** | **Comment** | **Response** | **Statement added to the body of manuscript** |
| --- | --- | --- | --- |
| **Editor Comments:** |  |  |  |
|  | 1. Inconsistency in study timeline The reported timeline requires clarification. The ethics approval number (KFU-REC-2024-OCT-ETHICS2653) indicates approval in October 2024, which aligns with the stated data collection period (October–November 2024). However, the  Methods section (Page 9, Line 7) states the study was conducted in May 2025. Please resolve this inconsistency and clearly distinguish between the periods of ethical approval, data collection, and data analysis. If the May 2025 date is an error, correct it. | We thank the Editor for this suggestion. Yes earlier it was not cited clearly. Now it is clearly updated and added into manuscript body. | Data were collected during the period from October 27^th^ to November 3^rd^ , 2024 and data analysis was conducted in may 2025. |
|  | 2. Sampling strategy and representativeness. The described sampling strategy requires further clarification. While a multi-stage sampling approach is reported, participant recruitment via social media platforms raises concerns about potential selection bias. Please:  • Clarify whether social media was used only to contact pre-selected individuals (from resident lists using fixed interval sampling) or whether participants could self-select via shared links. If the latter, this constitutes convenience sampling and the manuscript must be revised accordingly.  • Report the number of individuals invited versus those who participated.  • Provide the response rate and describe handling of non-response.  • Discuss potential selection and non-response biases in the limitations section.  • Revise claims of "representative sampling" if not fully supported by the recruitment method. | We appreciate the Editor’s request for clarification.  Social media was used just as a communication tool to contact pre-selected individuals identified through a multi stage sampling procedure, specifically using fixed-interval selection from the resident lists. After participants were selected, they were contacted through the available means of communication such as: social media platforms such as (WhatsApp, Telegram, Snapchat), and mobile phones to schedule an interview, for completing the questionnaire. No open links were shared, and participants could not self-select into the study. This approach ensured that the sampling remained structured and systematic. We have clarified this in the Methods section (Page 4: Sample technique : Method Section line 30 -50 ) |  |
|  | 3. Measurement and validation of the stigma construct (Critical) .  The study relies on a modified questionnaire derived from existing scales. However, the authors reworded items, reduced the scale to 5 questions, created a new composite scoring system, and applied an arbitrary mean cutoff (≥5). This constitutes a novel instrument, not a validated scale. Cronbach's alpha (0.83) measures internal consistency but does not establish construct validity.Given that the primary exposure variable (stigma) and the main prevalence estimate (23.1%) are derived from this unvalidated measure, additional validation is required.The authors must:􀀀 Conduct Exploratory Factor Analysis (EFA) and Confirmatory Factor Analysis (CFA) on the full sample (N=1085), which is more than sufficient for these procedures.􀀀 Report factor loadings, explained variance, and model fit indices (CFI, RMSEA, SRMR).􀀀 Re-evaluate the cutoff point empirically (e.g., using factor scores or ROC analysis) rather than relying on the mean.􀀀 Re-run all prevalence estimates and regression analyses using the validated scoring approach.􀀀 Clarify whether the scale is intended as a validated instrument or a composite indicator and discuss measurement limitations transparently. If the authors choose not to perform factor analysis, they must provide a compelling methodological justification — though such justification is unlikely to satisfy PLOS ONE's requirement for technical soundness | We appreciate the editor's clarification regarding measurement and validation requirements. While we previously addressed this by stating the absence of factor analysis in our limitations section, we recognize the importance of technical soundness as per PLOS ONE guidelines. So, we have revised the 'Instruments and Methods' section (Page 5, lines 21–32) to provide a more rigorous methodological justification. We hope this clarification addresses the requirement for technical validation. | The questionnaire was developed based on the key concepts derived from " Link’s Devaluation–Discrimination Scale" (13), and" Self-Stigma of Mental Illness Scale" (51), specifically related to the variables concerning to stigma and help-seeking in addressing psychological challenges. Some items rephrased to align with the linguistic and educational level of the study population, as well as to minimize response time. Although factor analysis was not conducted, content validity was ensured through a combination of rigorous literature review, consultation with public health specialist and focus group discussions. Based on their feedback, certain items were modified or removed to improve clarity and relevance based on their feedback. The scale was intended as a composite indicator of stigma rather than a fully validated psychometric instrument. To assess the reliability of the questionnaire, a pilot study was conducted on a random sample from the study population. Cronbach's alpha was calculated. The results reveal the questionnaire demonstrate high validity, with a Cronbach's alpha equal (0. 83). |
| **Minor Comments** |  |  |  |
|  | 1. Language and clarity. The manuscript would benefit from careful language editing to improve clarity and readability. Minor grammatical errors and awkward phrasing are present throughout. | Addressed and checked throughout manuscript. |  |
|  | 2. Terminology consistency Correct "sigma" to "stigma" where it appears (e.g., Page 9, Line 28). | Corrected as suggested |  |
|  | 3. Interpretation of findings. Avoid causal language when discussing associations due to the cross-sectional design (e.g., revise "stigma reduces help-seeking" to "stigma is associated with lower help-seeking"). | Corrected as suggested |  |
|  | Data availability statement􀀀 The Figshare dataset (DOI: 10.6084/m9.figshare.31811980.v2) appears to include only SPSS files. Please clarify whether the data are provided in a format accessible without proprietary software (e.g., CSV or Excel) to ensure full compliance with PLOS ONE data availability and reproducibility requirements.􀀀 | Yes, the data is openly accessible through the Figshare repository. We are also providing the data in Excel format to make it easier for reader’s easy accessibility as a supplementary file through PLOS one system. |  |
|  | Remove the duplicate "Informed Consent Statement" on Page 20 | Thanks for editor to mention it, revised accordingly. |  |
| Reviewer #1 |  |  |  |
|  | Dear Authors,  Thank you for the opportunity to re-review your manuscript. You have addressed the questions I raised, and I appreciate your responses. | We appreciate reviewer’s response to enhance the quality of manuscript. |  |
|  | However, a few issues remain:  Line 29 is missing a period. | Corrected. |  |
|  | The paragraph structure throughout the manuscript is still inadequate and requires improvement. In Table 3, the bottom horizontal line is missing. | Checked |  |
