# Supplementary material for: Mental illness stigma and its impact on help-seeking behavior among residents of the Eastern Region, Saudi Arabia: A cross-sectional study
Source: PLoS One. 2026 Jun 24;21(6):e0350860. doi: 10.1371/journal.pone.0350860 (PMC13293441; doi:10.1371/journal.pone.0350860)
Supplement: Attachment — Submitted filename: Response to reviewers R5.docx [file pone.0350860.s009.docx]

**Response to Editorial team and reviewer’s**

| **Sr. No.** | **Comment** | **Response** | **Statement added to the body of manuscript** |
| --- | --- | --- | --- |
| **Editor Comments:** | I invite the authors to:  * Conduct and report EFA/CFA on the current sample to validate the stigma scale, or* Provide a much stronger methodological justification for treating the scale as a composite indicator, including empirical justification for the cutoff (e.g., median split or ROC analysis) and re-running the main analysis with sensitivity checks. Without addressing this, the validity of the core findings remains questionable. | We thank the editor for this constructive suggestion. We conducted a factor analysis of the five-item stigma scale. | The questionnaire was developed based on the key concepts derived from " Link’s Devaluation–Discrimination Scale" (13), and" Self-Stigma of Mental Illness Scale" (51), specifically related to the variables concerning to stigma and help-seeking in addressing psychological challenges. Some items rephrased to align with the linguistic and educational level of the study population, as well as to minimize response time. The content validity was ensured through a combination of rigorous literature review, consultation with public health specialist and focus group discussions. Based on their feedback, certain items were modified or removed to improve clarity and relevance based on their feedback. Moreover, a factor analysis was conducted, of the five item stigma scale (N= 1085) using principal component analysis with Varimax rotation. The KMO measure was 0.491 and Bartlett’s test was significant (p < 0.001) supporting factorability. Communalities ranged from 0.27 to 0.64, indicating adequate item contribution. |
|  | Additionally, please report the response rate and add non-response bias to the limitations. | Thanks to editor.  A statement is added in the limitation section as suggested. | which may be subject to recall bias, social desirability bias, and the study subjected to non-response bias, which may limit the accuracy and generalizability of the finding. |
