# Supplementary material for: Mental illness stigma and its impact on help-seeking behavior among residents of the Eastern Region, Saudi Arabia: A cross-sectional study
Source: PLoS One. 2026 Jun 24;21(6):e0350860. doi: 10.1371/journal.pone.0350860 (PMC13293441; doi:10.1371/journal.pone.0350860)
Supplement: Attachment — Submitted filename: Response to reviewers R6.docx [file pone.0350860.s010.docx]

**Response to Editorial team and reviewer’s**

| **Sr. No.** | **Comment** | **Response** | **Statement added to the body of manuscript** |
| --- | --- | --- | --- |
| **Editor Additional Comments:** | Please report the number of eligible individuals who were contacted and the calculated response rate (%). If this information was not recorded, please state this as a limitation and justify why it could not be calculated. | We thank the editor for their additional suggestions to improve the quality and we updated our manuscript as required in:  Method section: Sample technique: Line 52. | A total of 1085 responses were collected, exceeding the minimum required sample size of 767.Among the eligible individuals who were contacted, six declined to participate, resulting in a non-response rate of 0.6%. |
