# Supplementary material for: Mental illness stigma and its impact on help-seeking behavior among residents of the Eastern Region, Saudi Arabia: A cross-sectional study
Source: PLoS One. 2026 Jun 24;21(6):e0350860. doi: 10.1371/journal.pone.0350860 (PMC13293441; doi:10.1371/journal.pone.0350860)
Supplement: Peer review history

# Peer review: Decision Letter 0

**DOI:** 10.1371/journal.pone.0350860.r001

**Authors:** Mohamed Said

20 Nov 2025

PONE-D-25-57473

Mental Illness Stigma and its impact on Help-Seeking Behavior among Residents of the Eastern Region, Saudi Arabia: A Cross-Sectional Study

PLOS ONE

Dear Dr. Ghazi I. Al Jowf,

Thank you for submitting your manuscript to PLOS ONE. After careful consideration, we feel that it has merit but does not fully meet PLOS ONE’s publication criteria as it currently stands. Therefore, we invite you to submit a revised version of the manuscript that addresses the points raised during the review process.

Please submit your revised manuscript within Jan 04 2026 11:59PM. If you will need more time than this to complete your revisions, please reply to this message or contact the journal office at plosone@plos.org. When you're ready to submit your revision, log on to https://www.editorialmanager.com/pone/ and select the 'Submissions Needing Revision' folder to locate your manuscript file.

Please include the following items when submitting your revised manuscript:

- A rebuttal letter that responds to each point raised by the academic editor and reviewer(s). You should upload this letter as a separate file labeled 'Response to Reviewers'.
- A marked-up copy of your manuscript that highlights changes made to the original version. You should upload this as a separate file labeled 'Revised Manuscript with Track Changes'.
- An unmarked version of your revised paper without tracked changes. You should upload this as a separate file labeled 'Manuscript'.

If you would like to make changes to your financial disclosure, please include your updated statement in your cover letter. Guidelines for resubmitting your figure files are available below the reviewer comments at the end of this letter.

If applicable, we recommend that you deposit your laboratory protocols in protocols.io to enhance the reproducibility of your results. Protocols.io assigns your protocol its own identifier (DOI) so that it can be cited independently in the future. For instructions see: https://journals.plos.org/plosone/s/submission-guidelines#loc-laboratory-protocols. Additionally, PLOS ONE offers an option for publishing peer-reviewed Lab Protocol articles, which describe protocols hosted on protocols.io. Read more information on sharing protocols at https://plos.org/protocols?utm_medium=editorial-email&utm_source=authorletters&utm_campaign=protocols.

We look forward to receiving your revised manuscript.

Kind regards,

Mohamed Ahmed Said, Ph.D.

Academic Editor

PLOS ONE

Journal Requirements:

When submitting your revision, we need you to address these additional requirements.

1. Please ensure that your manuscript meets PLOS ONE's style requirements, including those for file naming. The PLOS ONE style templates can be found at

https://journals.plos.org/plosone/s/file?id=wjVg/PLOSOne_formatting_sample_main_body.pdf and

https://journals.plos.org/plosone/s/file?id=ba62/PLOSOne_formatting_sample_title_authors_affiliations.pdf.

2. In the online submission form, you indicated that [All relevant data are within the manuscript, supplementary data can be provided on request.].

All PLOS journals now require all data underlying the findings described in their manuscript to be freely available to other researchers, either 1. In a public repository, 2. Within the manuscript itself, or 3. Uploaded as supplementary information.

This policy applies to all data except where public deposition would breach compliance with the protocol approved by your research ethics board. If your data cannot be made publicly available for ethical or legal reasons (e.g., public availability would compromise patient privacy), please explain your reasons on resubmission and your exemption request will be escalated for approval.

3. Please be informed that funding information should not appear in the Acknowledgments section or other areas of your manuscript. We will only publish funding information present in the Funding Statement section of the online submission form. Please remove any funding-related text from the manuscript.

4. Your ethics statement should only appear in the Methods section of your manuscript. If your ethics statement is written in any section besides the Methods, please delete it from any other section.

5. If the reviewer comments include a recommendation to cite specific previously published works, please review and evaluate these publications to determine whether they are relevant and should be cited. There is no requirement to cite these works unless the editor has indicated otherwise.

[Note: HTML markup is below. Please do not edit.]

Reviewers' comments:

Reviewer's Responses to Questions

**Comments to the Author**

1. Is the manuscript technically sound, and do the data support the conclusions?

The manuscript must describe a technically sound piece of scientific research with data that supports the conclusions. Experiments must have been conducted rigorously, with appropriate controls, replication, and sample sizes. The conclusions must be drawn appropriately based on the data presented.

Reviewer #1: Yes

Reviewer #2: Partly

**********

2. Has the statistical analysis been performed appropriately and rigorously?

Reviewer #1: Yes

Reviewer #2: No

**********

3. Have the authors made all data underlying the findings in their manuscript fully available?

The PLOS Data policy requires authors to make all data underlying the findings described in their manuscript fully available without restriction, with rare exception (please refer to the Data Availability Statement in the manuscript PDF file). The data should be provided as part of the manuscript or its supporting information, or deposited to a public repository. For example, in addition to summary statistics, the data points behind means, medians and variance measures should be available. If there are restrictions on publicly sharing data—e.g. participant privacy or use of data from a third party—those must be specified.

Reviewer #1: Yes

Reviewer #2: No

**********

4. Is the manuscript presented in an intelligible fashion and written in standard English?

PLOS ONE does not copyedit accepted manuscripts, so the language in submitted articles must be clear, correct, and unambiguous. Any typographical or grammatical errors should be corrected at revision, so please note any specific errors here.

Reviewer #1: Yes

Reviewer #2: No

**********

5. Review Comments to the Author

Please use the space provided to explain your answers to the questions above. You may also include additional comments for the author, including concerns about dual publication, research ethics, or publication ethics. (Please upload your review as an attachment if it exceeds 20,000 characters)

Reviewer #1: Thanks for this interesting paper to review,The content of this manuscript is relevant to the scope of PLOS. The study addresses a critical public health issue, mental illness stigma and its impact on help-seeking behavior. With 1085 participants, the study has strong statistical power and allows for meaningful subgroup analysis. The use of descriptive statistics, chi-square tests, and logistic regression provides a solid framework to explore associations and predictors.

Reviewer #2: The topic is relevant to public health and aligns with the aims of PLOS ONE, addressing stigma and mental health service utilization in a Middle Eastern context that remains under-researched. However, substantial revisions are required to meet the journal’s methodological and reporting standards.

General Assessment

The study addresses an important issue and contributes potentially valuable regional data to the global literature on mental illness stigma and help-seeking behavior. The manuscript demonstrates commendable effort in sample recruitment and descriptive reporting. Nevertheless, the current version requires significant improvement in conceptual framing, methodological rigor, data interpretation, and formal presentation. The design, though appropriate in general terms, lacks validation details of the measurement tools and shows inconsistencies between the statistical analysis and the conclusions drawn.

Overall, I recommend Major Revision before the paper can be considered for publication.

Evaluation According to PLOS ONE Criteria

1. The study presents the results of original research

The study appears original and has not been previously published. The topic is timely and underexplored in the Saudi context. However, the authors should explicitly state in the introduction what gap in the existing literature their work addresses and how it contributes beyond descriptive regional data.

2. Results reported have not been published elsewhere

There is no indication that the results were published elsewhere;

this criterion is met.

3. Experiments, statistics, and other analyses are performed to a high technical standard and are described in sufficient detail.

The statistical analyses (chi-square and logistic regression) are appropriate in principle, but the operationalization of key variables is insufficiently justified.

The self-developed stigma and help-seeking scales lack validation against recognized instruments (e.g., Link’s Devaluation–Discrimination Scale, or the Self-Stigma of Mental Illness Scale).

The reported “response rate of 141.5%” is a critical methodological error and must be corrected or clarified.

No description of multicollinearity checks or model fit diagnostics is provided for logistic regression.

Dichotomization thresholds (e.g., “<5 indicates no stigma”) are arbitrary and require theoretical or empirical justification.

This criterion is unmet

4. Conclusions are presented in an appropriate fashion and are supported by the data.

Conclusions are generally consistent with the data, but they are overstated. For instance, labeling the prevalence of stigma as “low” is not justified without comparative, methodologically similar data. The claim that “young adults and females are more likely to seek help” should be discussed in a socio-cultural context rather than simply restating numerical findings. It requires significant revision for interpretative depth and caution.

5. The article is presented in an intelligible fashion and is written in standard English

The manuscript is intelligible but contains numerous grammatical, syntactic, and stylistic errors.

o Subject–verb disagreement (“The study indicate…” → “The study indicates”).

o Inconsistent terminology (“mental illness stigma”, “mental health stigma”).

o Redundant phrasing and repetition throughout.

Requires thorough language editing by a native or professional scientific editor.

6. The research meets all applicable standards for the ethics of experimentation and research integrity.

Ethical approval and informed consent are appropriately stated. However, the Data Availability section is inconsistent: the form declares “data are fully available”, while the manuscript says “available upon reasonable request”. PLOS ONE requires unrestricted data availability. This inconsistency must be resolved.

Ethical criteria met; data transparency not yet compliant.

7. The article adheres to appropriate reporting guidelines and community standards for data availability.

The paper lacks adherence to established reporting guidelines for cross-sectional studies (e.g., STROBE).

Tables are clear but lack clear variable definitions and labeling consistency. The instrument development process should be described in detail, including item examples and scoring rationale.

Partial compliance; improvement needed for transparency and replicability.

Major Comments

1. Conceptual Framing

The introduction should include a stronger theoretical grounding in stigma research (e.g., Corrigan & Watson’s framework; Link & Phelan’s model). The current framing is descriptive rather than analytical.

Corrigan PW, Watson AC. Understanding the impact of stigma on people with mental illness. World Psychiatry. 2002 Feb;1(1):16-20. PMID: 16946807; PMCID: PMC1489832.

Link, B. G., & Phelan, J. C. (2001). Conceptualizing Stigma. Annual Review of Sociology, 27, 363–385. http://www.jstor.org/stable/2678626

2. Instrument Validation

Provide detailed information on how the stigma and help-seeking scales were developed, tested, and validated. If they are novel, include factor analysis and reliability indices beyond Cronbach’s alpha.

3. Statistical Analysis

Explain the rationale for the dichotomization of stigma and help-seeking scores. Consider reanalyzing the data using continuous or ordinal scales to preserve statistical power.

4. Interpretation of Results

The discussion section should distinguish between association and causation. Statements implying that stigma “leads to” help-seeking avoidance should be rephrased to reflect a correlational design.

5. Comparative Context

Avoid direct comparisons with prevalence rates from other countries unless similar instruments and populations were used. Instead, emphasize contextual explanations (e.g., cultural norms, healthcare infrastructure).

6. Data Transparency

Make all de-identified data and the full questionnaire publicly available (e.g., via a repository link).

7. Language and Readability

The manuscript needs comprehensive professional English editing to meet publication standards.

Minor Comments

1. Check consistency of numerical data between abstract, results, and tables.

2. Correct typographical and formatting inconsistencies (e.g., decimal points, percentage spacing).

3. Ensure all references follow PLOS ONE style (complete DOI, correct capitalization).

4. Revise phrases like “the study indicate that” and “vice versa” for clarity and conciseness.

5. Replace “low” or “high” with quantitative descriptors where possible (e.g., “23.1%, lower than in prior regional studies”).

6. Verify that the ethical approval number matches the institutional record.

**********

6. PLOS authors have the option to publish the peer review history of their article (what does this mean?). If published, this will include your full peer review and any attached files.

If you choose “no”, your identity will remain anonymous but your review may still be made public.

**Do you want your identity to be public for this peer review?**     For information about this choice, including consent withdrawal, please see our Privacy Policy.

Reviewer #1: No

Reviewer #2: **Yes:**     Elisa Caselani

**********

[NOTE: If reviewer comments were submitted as an attachment file, they will be attached to this email and accessible via the submission site. Please log into your account, locate the manuscript record, and check for the action link "View Attachments". If this link does not appear, there are no attachment files.]

To ensure your figures meet our technical requirements, please review our figure guidelines: https://journals.plos.org/plosone/s/figures

You may also use PLOS’s free figure tool, NAAS, to help you prepare publication quality figures: https://journals.plos.org/plosone/s/figures#loc-tools-for-figure-preparation.

NAAS will assess whether your figures meet our technical requirements by comparing each figure against our figure specifications.

Attachment Submitted filename: Review of Manuscript_PLOS 251108.docx

# Author comment: Author response to Decision Letter 1

**DOI:** 10.1371/journal.pone.0350860.r002

17 Feb 2026

We have addressed all the comments provided by the Editor and the reviewers. Our detailed point-by-point responses are included in the attached 'Response to Reviewers' file, and the manuscript has been revised accordingly.

Thank you for your time and consideration.

Attachment Submitted filename: Response to Reviewers 17-02-2026.docx

# Peer review: Decision Letter 1

**DOI:** 10.1371/journal.pone.0350860.r003

**Authors:** Mohamed Said

2 Mar 2026

PONE-D-25-57473R1

Mental Illness Stigma and its impact on Help-Seeking Behavior among Residents of the Eastern Region, Saudi Arabia: A Cross-Sectional Study

PLOS One

Dear Dr. Al Jowf,

Thank you for submitting your manuscript to PLOS ONE. After careful consideration, we feel that it has merit but does not fully meet PLOS ONE’s publication criteria as it currently stands. Therefore, we invite you to submit a revised version of the manuscript that addresses the points raised during the review process.

Please submit your revised manuscript by Apr 16 2026 11:59PM. If you will need more time than this to complete your revisions, please reply to this message or contact the journal office at plosone@plos.org. When you're ready to submit your revision, log on to https://www.editorialmanager.com/pone/ and select the 'Submissions Needing Revision' folder to locate your manuscript file.

Please include the following items when submitting your revised manuscript:

- A letter that responds to each point raised by the academic editor and reviewer(s). You should upload this letter as a separate file labeled 'Response to Reviewers'.
- A marked-up copy of your manuscript that highlights changes made to the original version. You should upload this as a separate file labeled 'Revised Manuscript with Track Changes'.
- An unmarked version of your revised paper without tracked changes. You should upload this as a separate file labeled 'Manuscript'.

If you would like to make changes to your financial disclosure, please include your updated statement in your cover letter. Guidelines for resubmitting your figure files are available below the reviewer comments at the end of this letter.

If applicable, we recommend that you deposit your laboratory protocols in protocols.io to enhance the reproducibility of your results. Protocols.io assigns your protocol its own identifier (DOI) so that it can be cited independently in the future. For instructions see: https://journals.plos.org/plosone/s/submission-guidelines#loc-laboratory-protocols. Additionally, PLOS ONE offers an option for publishing peer-reviewed Lab Protocol articles, which describe protocols hosted on protocols.io. Read more information on sharing protocols at https://plos.org/protocols?utm_medium=editorial-email&utm_source=authorletters&utm_campaign=protocols.

We look forward to receiving your revised manuscript.

Kind regards,

Mohamed Ahmed Said, Ph.D.

Academic Editor

PLOS One

**Journal Requirements:**

If the reviewer comments include a recommendation to cite specific previously published works, please review and evaluate these publications to determine whether they are relevant and should be cited. There is no requirement to cite these works unless the editor has indicated otherwise.

[Note: HTML markup is below. Please do not edit.]

Reviewers' comments:

Reviewer's Responses to Questions

**Comments to the Author**

1. If the authors have adequately addressed your comments raised in a previous round of review and you feel that this manuscript is now acceptable for publication, you may indicate that here to bypass the “Comments to the Author” section, enter your conflict of interest statement in the “Confidential to Editor” section, and submit your "Accept" recommendation.

Reviewer #1: All comments have been addressed

Reviewer #2: (No Response)

**********

2. Is the manuscript technically sound, and do the data support the conclusions?

The manuscript must describe a technically sound piece of scientific research with data that supports the conclusions. Experiments must have been conducted rigorously, with appropriate controls, replication, and sample sizes. The conclusions must be drawn appropriately based on the data presented.

Reviewer #1: Yes

Reviewer #2: Partly

**********

3. Has the statistical analysis been performed appropriately and rigorously?

Reviewer #1: Yes

Reviewer #2: No

**********

4. Have the authors made all data underlying the findings in their manuscript fully available?

The PLOS Data policy requires authors to make all data underlying the findings described in their manuscript fully available without restriction, with rare exception (please refer to the Data Availability Statement in the manuscript PDF file). The data should be provided as part of the manuscript or its supporting information, or deposited to a public repository. For example, in addition to summary statistics, the data points behind means, medians and variance measures should be available. If there are restrictions on publicly sharing data—e.g. participant privacy or use of data from a third party—those must be specified.

Reviewer #1: No

Reviewer #2: No

**********

5. Is the manuscript presented in an intelligible fashion and written in standard English?

PLOS ONE does not copyedit accepted manuscripts, so the language in submitted articles must be clear, correct, and unambiguous. Any typographical or grammatical errors should be corrected at revision, so please note any specific errors here.

Reviewer #1: No

Reviewer #2: No

**********

6. Review Comments to the Author

Please use the space provided to explain your answers to the questions above. You may also include additional comments for the author, including concerns about dual publication, research ethics, or publication ethics. (Please upload your review as an attachment if it exceeds 20,000 characters)

**Reviewer #1:**     It is difficult to thoroughly review the manuscript in its current form due to the absence of line numbers and insufficient paragraph structure. Adding line numbers and improving the paragraph division would greatly facilitate a more precise and constructive review and make it easier for the reader.

References vary, sometimes tendency of APA 7 and sometimes tendency of Vancouver, this need to be checked out. In text another reference system.

The PLOS Data policy requires authors to make all data underlying the findings described in their manuscript fully available without restriction,as I can see you write All relevant data are within the manuscript, supplementary data can be provided on request, where is all data, it is only the analysis provided in the paper.

**Reviewer #2:**     Re: Second Review - Response to Authors’ Replies

Manuscript: Mental Illness Stigma and its Impact on Help-Seeking Behavior among Residents of the Eastern Region, Saudi Arabia: A Cross-Sectional Study

Journal: PLOS ONE

Dear Editor,

I thank the authors for their revised manuscript and their point-by-point responses to the first round of comments. I acknowledge that several concerns have been adequately addressed, including expanding the theoretical framework in the introduction (Corrigan & Watson; Link & Phelan), addressing the socio-cultural interpretation of gender and age differences in help-seeking, and revising the language and formatting.

However, several substantive methodological issues remain insufficiently resolved. I outline these below, distinguishing between points that I consider satisfactorily addressed, those that require minor revision, and those that represent major outstanding concerns.

1. Points Considered Satisfactorily Addressed

The following points from the first round have been addressed to a satisfactory degree and require no further revision:

• 3.4 (multicollinearity check), 5.1–5.3 (language corrections), 6.1 (ethical approval), 7.1 (STROBE alignment), 7.2 (table corrections), 8.1 (theoretical grounding), 11 (causation vs. correlation language), 15–20 (minor corrections).

2. Major Outstanding Concerns

Point 3.2 and 9.1 and 9.2 - Instrument Validation (MAJOR, UNRESOLVED)

Original concern: The self-developed stigma and help-seeking scales lack validation against recognized instruments. Factor analysis and additional reliability indices were requested.

The authors’ response acknowledges the absence of formal validation but argues that conceptual alignment with Link's Devaluation–Discrimination Scale and the Self-Stigma of Mental Illness Scale is sufficient. This is not acceptable for a PLOS ONE submission. Conceptual alignment during item generation is a preliminary step, not a substitute for psychometric validation.

Specifically:

(a) The claim that the questionnaire is “not a novel scale” and therefore does not require factor analysis is incorrect. Any self-developed instrument, regardless of its conceptual origins, requires structural validation particularly when used to produce prevalence estimates that form the primary outcome of the study.

(b) Cronbach’s alpha (0.83) measures internal consistency but provides no evidence of construct validity, dimensionality, or criterion-related validity.

(c) The revised manuscript should either: (i) report an exploratory or confirmatory factor analysis to establish the dimensional structure of the instrument; or (ii) include an explicit acknowledgment in the Limitations section that the absence of formal psychometric validation is a significant constraint on the generalizability and comparability of findings. This limitation must be discussed substantively, not simply mentioned in passing.

Point 3.5 and 10.1 - Dichotomization Thresholds (MAJOR, PARTIALLY RESOLVED)

Original concern: Dichotomization thresholds are arbitrary. Reanalysis using continuous or ordinal scales was recommended.

The authors justify using the mean as a cutoff by citing two previous studies (Lapham et al., 2022; Hadera et al., 2019). This justification remains weak. Neither reference appears to use a comparable instrument or study design, and citing precedent for a practice does not resolve its methodological limitations.

Furthermore, note that the response document itself contains an internal contradiction: under Point 3.5, it states both 'Mean score ≥ 5 indicates no mental illness-related stigma' AND 'indicates presence of mental illness-related stigma', these are mutually exclusive and suggest the revision was not reviewed carefully.

Furthermore, the Methods section (p. 12) contains another logical contradiction in the definition of the help-seeking cutoff that was not present in the response letter but appears in the revised manuscript: “a score of less than 3 indicates seeking help and a score <3 indicates being deterred from seeking help”. Both conditions use the same threshold (<3) to define two mutually exclusive outcomes, which is logically impossible and renders the operationalization of the outcome variable unintelligible.

The authors should at minimum: (i) correct the internal contradictions noted above in both the response document and the manuscript; (ii) provide a more substantive theoretical or empirical rationale for the specific cutoff of 5 (not simply that the mean was used); (iii) acknowledge in the Limitations section that dichotomization leads to loss of information and may inflate or deflate prevalence estimates depending on the distribution of scores.

Point 6.2 and 13 - Data Availability (UNRESOLVED, POLICY VIOLATION)

This issue was raised in the first round of review and remains unresolved. The submission form contains a direct contradiction: the authors selected “Yes - all data are fully available without restriction” but then wrote in the text box below: “supplementary data can be provided on request”. These two statements are mutually exclusive. Additionally, the Data Availability statement in the manuscript (p. 13) states that data are "available as supplementary file" without providing any repository link, DOI, or accession number.

PLOS ONE’s data availability policy is unambiguous: data must be fully and unconditionally available at the time of publication, without requiring readers to contact the authors. Stating that data are available "on request" or "as a supplementary file" without a retrievable location does not meet this requirement.

To comply, the authors must take one of the following actions before resubmission:

1.

Deposit the full de-identified dataset in a recognized public repository (e.g., Figshare, Zenodo, OSF, or Dryad), obtain a DOI, and include it in the Data Availability Statement; or

2.

Upload the dataset as a formal supplementary file through the journal submission system, ensuring it is publicly accessible upon publication - not upon request.

The Data Availability Statement in the manuscript must then be updated to reflect exactly where and how the data can be accessed, with a direct link or DOI. Failure to resolve this point may result in rejection on editorial policy grounds, independent of the scientific merit of the study.

Adjunctive comments not previously recommended (MAJOR)

Critical Statistical Error: Significance Threshold (UNRESOLVED, PRESENT THROUGHOUT THE MANUSCRIPT)

A systematic typographical error appears repeatedly throughout the manuscript and has not been corrected in this revision. In Tables 2, 3, and 4, in the Results section, and in the Abstract, the significance threshold is consistently reported as “p < 0.5” instead of the correct “p < 0.05”. The authors must conduct a thorough search of the entire manuscript including the abstract, all tables, all figure legends, and the results and discussion sections and replace every instance of “p < 0.5” with the correct threshold “p < 0.05”. The authors should also verify that all reported p-values in the tables are consistent with this corrected threshold and that no conclusions change as a result.

This error must be corrected before the manuscript can be considered for publication.

3. Minor Remaining Concerns

Point 3.3 - Response Rate (STILL REQUIRES CORRECTION IN THE MANUSCRIPT)

The authors' explanation in the response letter is acceptable: collecting more responses than the minimum required sample size is a legitimate methodological decision, and including all valid responses in the analysis is appropriate. However, the manuscript itself (Methods section, p. 10) still reads: “A total of 1085 responses were interviewed, yielding a response rate of 141.5%”.This sentence must be corrected in the manuscript text, as a response rate exceeding 100% is a formal error that cannot appear in a published article, regardless of the explanation provided separately in the response letter.

The authors should remove the response rate figure entirely and replace it with a sentence along the following lines: “A total of 1085 responses were collected, exceeding the minimum required sample size of 767. This was due to higher-than-expected participation in Al-Hofuf and Al-Qura. All responses were verified for completeness and validity and were included in the final analysis”.

Point 4.1 - Comparative Prevalence Framing (MINOR)

Original concern: Labeling stigma prevalence as 'low' without methodologically comparable data.

The authors have added comparative figures from other countries but acknowledge in Point 12 that different instruments and populations were used. The revised text should include a clear caveat whenever cross-national comparisons are made, explicitly noting that differences in instruments, sampling frames, and cultural contexts limit direct comparability. A sentence to this effect should be added each time comparative data are cited in the Discussion.

Point 5.1 - Typographical Errors in the Abstract (DECLARED AS CORRECTED, STILL PRESENT)

The authors stated in their response to Point 5.1 that subject-verb disagreement had been revised throughout the manuscript. However, the abstract (p. 7) still contains two uncorrected errors. First, “The study indicate” should read “The study indicates”. Second, the prevalence figure is reported as “23.1” without the percentage symbol, which should read “23.1%”. Both errors remain in the revised manuscript despite the authors explicitly declaring them as corrected. The authors must carefully proofread the abstract in its entirety, as these are the first sentences a reader encounters and their presence undermines the overall quality of the manuscript.

Point 12 - Cross-National Comparisons (MINOR)

Original concern: Direct comparisons with other countries using different instruments are methodologically problematic.

The authors’ response states they are “showing a trend”, but the manuscript does not adequately distinguish between trend-level observations and direct comparisons. This conflation risks misleading readers. I recommend the authors revise the relevant passages to use language such as 'rates appear higher/lower than those reported in studies from X, though direct comparison is limited by differences in measurement instruments and study populations.'

4. Summary Recommendation

In its current form, the manuscript cannot be recommended for acceptance. The four major concerns identified above: instrument validation, dichotomization justification, and data availability, must be fully resolved before the manuscript can be considered further. I encourage the authors to address these points thoroughly in the next revision.

I remain willing to review a further revised version of the manuscript.

**********

7. PLOS authors have the option to publish the peer review history of their article (what does this mean?). If published, this will include your full peer review and any attached files.

If you choose “no”, your identity will remain anonymous but your review may still be made public.

**Do you want your identity to be public for this peer review?**     For information about this choice, including consent withdrawal, please see our Privacy Policy.

Reviewer #1: No

Reviewer #2: **Yes:**     Elisa Caselani

**********

[NOTE: If reviewer comments were submitted as an attachment file, they will be attached to this email and accessible via the submission site. Please log into your account, locate the manuscript record, and check for the action link "View Attachments". If this link does not appear, there are no attachment files.]

To ensure your figures meet our technical requirements, please review our figure guidelines: https://journals.plos.org/plosone/s/figures

You may also use PLOS’s free figure tool, NAAS, to help you prepare publication quality figures: https://journals.plos.org/plosone/s/figures#loc-tools-for-figure-preparation.

NAAS will assess whether your figures meet our technical requirements by comparing each figure against our figure specifications.

Attachment Submitted filename: Review 260226 PLOS one.docx

Attachment Submitted filename: Second_Response_to_Authors_Stigma_PLOS.pdf

# Author comment: Author response to Decision Letter 2

**DOI:** 10.1371/journal.pone.0350860.r004

4 Apr 2026

Please find attached along with the revised manuscript.

Attachment Submitted filename: Response_R2_04_04_26.docx

# Peer review: Decision Letter 2

**DOI:** 10.1371/journal.pone.0350860.r005

**Authors:** Mohamed Said

15 Apr 2026

PONE-D-25-57473R2

Mental Illness Stigma and its impact on Help-Seeking Behavior among Residents of the Eastern Region, Saudi Arabia: A Cross-Sectional Study

PLOS One

Dear Dr. Al Jowf,

Thank you for submitting your manuscript to PLOS ONE. After careful consideration, we feel that it has merit but does not fully meet PLOS ONE’s publication criteria as it currently stands. Therefore, we invite you to submit a revised version of the manuscript that addresses the points raised during the review process.

Please submit your revised manuscript by May 30 2026 11:59PM. If you will need more time than this to complete your revisions, please reply to this message or contact the journal office at plosone@plos.org. When you're ready to submit your revision, log on to https://www.editorialmanager.com/pone/ and select the 'Submissions Needing Revision' folder to locate your manuscript file.

Please include the following items when submitting your revised manuscript:

- A letter that responds to each point raised by the academic editor and reviewer(s). You should upload this letter as a separate file labeled 'Response to Reviewers'.
- A marked-up copy of your manuscript that highlights changes made to the original version. You should upload this as a separate file labeled 'Revised Manuscript with Track Changes'.
- An unmarked version of your revised paper without tracked changes. You should upload this as a separate file labeled 'Manuscript'.

If you would like to make changes to your financial disclosure, please include your updated statement in your cover letter. Guidelines for resubmitting your figure files are available below the reviewer comments at the end of this letter.

If applicable, we recommend that you deposit your laboratory protocols in protocols.io to enhance the reproducibility of your results. Protocols.io assigns your protocol its own identifier (DOI) so that it can be cited independently in the future. For instructions see: https://journals.plos.org/plosone/s/submission-guidelines#loc-laboratory-protocols. Additionally, PLOS ONE offers an option for publishing peer-reviewed Lab Protocol articles, which describe protocols hosted on protocols.io. Read more information on sharing protocols at https://plos.org/protocols?utm_medium=editorial-email&utm_source=authorletters&utm_campaign=protocols.

As the corresponding author, your ORCID iD is verified in the submission system and will appear in the published article. PLOS supports the use of ORCID, and we encourage all coauthors to register for an ORCID iD and use it as well. Please encourage your coauthors to verify their ORCID iD within the submission system before final acceptance, as unverified ORCID iDs will not appear in the published article. *Only*     the individual author can complete the verification step; PLOS staff *cannot*     verify ORCID iDs on behalf of authors.

We look forward to receiving your revised manuscript.

Kind regards,

Mohamed Ahmed Said, Ph.D.

Academic Editor

PLOS One

Journal Requirements:

If the reviewer comments include a recommendation to cite specific previously published works, please review and evaluate these publications to determine whether they are relevant and should be cited. There is no requirement to cite these works unless the editor has indicated otherwise.

Additional Editor Comments:

The manuscript addresses a relevant topic and has several strengths; however, the methodological concerns—particularly regarding sampling and measurement—need to be addressed to ensure the robustness and credibility of the findings.

1. Inconsistency in study timeline

The reported timeline requires clarification. The ethics approval number (KFU-REC-2024-OCT-ETHICS2653) indicates approval in October 2024, which aligns with the stated data collection period (October–November 2024). However, the Methods section (Page 9, Line 7) states the study was conducted in May 2025. Please resolve this inconsistency and clearly distinguish between the periods of ethical approval, data collection, and data analysis. If the May 2025 date is an error, correct it.

2. Sampling strategy and representativeness

The described sampling strategy requires further clarification. While a multi-stage sampling approach is reported, participant recruitment via social media platforms raises concerns about potential selection bias.

Please:

• Clarify whether social media was used only to contact pre-selected individuals (from resident lists using fixed interval sampling) or whether participants could self-select via shared links. If the latter, this constitutes convenience sampling and the manuscript must be revised accordingly.

• Report the number of individuals invited versus those who participated.

• Provide the response rate and describe handling of non-response.

• Discuss potential selection and non-response biases in the limitations section.

• Revise claims of "representative sampling" if not fully supported by the recruitment method.

3. Measurement and validation of the stigma construct (Critical)

The study relies on a modified questionnaire derived from existing scales. However, the authors reworded items, reduced the scale to 5 questions, created a new composite scoring system, and applied an arbitrary mean cutoff (≥5). This constitutes a novel instrument, not a validated scale. Cronbach's alpha (0.83) measures internal consistency but does not establish construct validity.

Given that the primary exposure variable (stigma) and the main prevalence estimate (23.1%) are derived from this unvalidated measure, additional validation is required.

The authors must:

Conduct Exploratory Factor Analysis (EFA) and Confirmatory Factor Analysis (CFA) on the full sample (N=1085), which is more than sufficient for these procedures.

Report factor loadings, explained variance, and model fit indices (CFI, RMSEA, SRMR).

Re-evaluate the cutoff point empirically (e.g., using factor scores or ROC analysis) rather than relying on the mean.

Re-run all prevalence estimates and regression analyses using the validated scoring approach.

Clarify whether the scale is intended as a validated instrument or a composite indicator and discuss measurement limitations transparently.

If the authors choose not to perform factor analysis, they must provide a compelling methodological justification — though such justification is unlikely to satisfy PLOS ONE's requirement for technical soundness.

Minor Comments

1. Language and clarity

The manuscript would benefit from careful language editing to improve clarity and readability. Minor grammatical errors and awkward phrasing are present throughout.

2. Terminology consistency

Correct "sigma" to "stigma" where it appears (e.g., Page 9, Line 28).

3. Interpretation of findings

Avoid causal language when discussing associations due to the cross-sectional design (e.g., revise "stigma reduces help-seeking" to "stigma is associated with lower help-seeking").

4. Data availability statement

The Figshare dataset (DOI: 10.6084/m9.figshare.31811980.v2) appears to include only SPSS files. Please clarify whether the data are provided in a format accessible without proprietary software (e.g., CSV or Excel) to ensure full compliance with PLOS ONE data availability and reproducibility requirements.

Remove the duplicate "Informed Consent Statement" on Page 20.

[Note: HTML markup is below. Please do not edit.]

Reviewers' comments:

Reviewer's Responses to Questions

**Comments to the Author**

1. If the authors have adequately addressed your comments raised in a previous round of review and you feel that this manuscript is now acceptable for publication, you may indicate that here to bypass the “Comments to the Author” section, enter your conflict of interest statement in the “Confidential to Editor” section, and submit your "Accept" recommendation.

Reviewer #1: All comments have been addressed

Reviewer #2: All comments have been addressed

**********

2. Is the manuscript technically sound, and do the data support the conclusions?

The manuscript must describe a technically sound piece of scientific research with data that supports the conclusions. Experiments must have been conducted rigorously, with appropriate controls, replication, and sample sizes. The conclusions must be drawn appropriately based on the data presented.

Reviewer #1: Yes

Reviewer #2: Yes

**********

3. Has the statistical analysis been performed appropriately and rigorously?

Reviewer #1: N/A

Reviewer #2: Yes

**********

4. Have the authors made all data underlying the findings in their manuscript fully available?

The PLOS Data policy requires authors to make all data underlying the findings described in their manuscript fully available without restriction, with rare exception (please refer to the Data Availability Statement in the manuscript PDF file). The data should be provided as part of the manuscript or its supporting information, or deposited to a public repository. For example, in addition to summary statistics, the data points behind means, medians and variance measures should be available. If there are restrictions on publicly sharing data—e.g. participant privacy or use of data from a third party—those must be specified.

Reviewer #1: Yes

Reviewer #2: Yes

**********

5. Is the manuscript presented in an intelligible fashion and written in standard English?

PLOS ONE does not copyedit accepted manuscripts, so the language in submitted articles must be clear, correct, and unambiguous. Any typographical or grammatical errors should be corrected at revision, so please note any specific errors here.

Reviewer #1: Yes

Reviewer #2: Yes

**********

6. Review Comments to the Author

Please use the space provided to explain your answers to the questions above. You may also include additional comments for the author, including concerns about dual publication, research ethics, or publication ethics. (Please upload your review as an attachment if it exceeds 20,000 characters)

Reviewer #1: Dear Authors,

Thank you for the opportunity to re-review your manuscript. You have addressed the questions I raised, and I appreciate your responses.

However, a few issues remain:

Line 29 is missing a period.

The paragraph structure throughout the manuscript is still inadequate and requires improvement.

In Table 3, the bottom horizontal line is missing.

Reviewer #2: (No Response)

**********

7. PLOS authors have the option to publish the peer review history of their article (what does this mean?). If published, this will include your full peer review and any attached files.

If you choose “no”, your identity will remain anonymous but your review may still be made public.

**Do you want your identity to be public for this peer review?**     For information about this choice, including consent withdrawal, please see our Privacy Policy.

Reviewer #1: No

Reviewer #2: **Yes:**     Elisa Caselani

**********

[NOTE: If reviewer comments were submitted as an attachment file, they will be attached to this email and accessible via the submission site. Please log into your account, locate the manuscript record, and check for the action link "View Attachments". If this link does not appear, there are no attachment files.]

To ensure your figures meet our technical requirements, please review our figure guidelines: https://journals.plos.org/plosone/s/figures

You may also use PLOS’s free figure tool, NAAS, to help you prepare publication quality figures: https://journals.plos.org/plosone/s/figures#loc-tools-for-figure-preparation.

NAAS will assess whether your figures meet our technical requirements by comparing each figure against our figure specifications.

# Author comment: Author response to Decision Letter 3

**DOI:** 10.1371/journal.pone.0350860.r006

27 Apr 2026

We attached a Response to Reviewers along with our updated manuscript.

Attachment Submitted filename: Response to reviewers 3rd.docx

# Editor report: Decision Letter 3

**DOI:** 10.1371/journal.pone.0350860.r007

**Authors:** Mohamed Said

1 May 2026

PONE-D-25-57473R3

Mental Illness Stigma and its impact on Help-Seeking Behavior among Residents of the Eastern Region, Saudi Arabia: A Cross-Sectional Study

PLOS One

Dear Dr. Al Jowf,

Thank you for submitting your manuscript to PLOS ONE. After careful consideration, we feel that it has merit but does not fully meet PLOS ONE’s publication criteria as it currently stands. Therefore, we invite you to submit a revised version of the manuscript that addresses the points raised during the review process.

Please submit your revised manuscript within Jun 15 2026 11:59PM from the date of this report. If you will need more time than this to complete your revisions, please reply to this message or contact the journal office at plosone@plos.org. When you're ready to submit your revision, log on to https://www.editorialmanager.com/pone/ and select the 'Submissions Needing Revision' folder to locate your manuscript file.

Please include the following items when submitting your revised manuscript:

- A letter that responds to each point raised by the academic editor and reviewer(s). You should upload this letter as a separate file labeled 'Response to Reviewers'.
- A marked-up copy of your manuscript that highlights changes made to the original version. You should upload this as a separate file labeled 'Revised Manuscript with Track Changes'.
- An unmarked version of your revised paper without tracked changes. You should upload this as a separate file labeled 'Manuscript'.

If you would like to make changes to your financial disclosure, please include your updated statement in your cover letter. Guidelines for resubmitting your figure files are available below the reviewer comments at the end of this letter.

If applicable, we recommend that you deposit your laboratory protocols in protocols.io to enhance the reproducibility of your results. Protocols.io assigns your protocol its own identifier (DOI) so that it can be cited independently in the future. For instructions see: https://journals.plos.org/plosone/s/submission-guidelines#loc-laboratory-protocols. Additionally, PLOS ONE offers an option for publishing peer-reviewed Lab Protocol articles, which describe protocols hosted on protocols.io. Read more information on sharing protocols at https://plos.org/protocols?utm_medium=editorial-email&utm_source=authorletters&utm_campaign=protocols.

As the corresponding author, your ORCID iD is verified in the submission system and will appear in the published article. PLOS supports the use of ORCID, and we encourage all coauthors to register for an ORCID iD and use it as well. Please encourage your coauthors to verify their ORCID iD within the submission system before final acceptance, as unverified ORCID iDs will not appear in the published article. *Only*     the individual author can complete the verification step; PLOS staff *cannot*     verify ORCID iDs on behalf of authors.

We look forward to receiving your revised manuscript.

Kind regards,

Mohamed Ahmed Said, Ph.D.

Academic Editor

PLOS One

Journal Requirements:

If the reviewer comments include a recommendation to cite specific previously published works, please review and evaluate these publications to determine whether they are relevant and should be cited. There is no requirement to cite these works unless the editor has indicated otherwise.

Please review your reference list to ensure that it is complete and correct. If you have cited papers that have been retracted, please include the rationale for doing so in the manuscript text, or remove these references and replace them with relevant current references. Any changes to the reference list should be mentioned in the rebuttal letter that accompanies your revised manuscript. If you need to cite a retracted article, indicate the article’s retracted status in the References list and also include a citation and full reference for the retraction notice.

Additional Editor Comments:

I invite the authors to:

* Conduct and report EFA/CFA on the current sample to validate the stigma scale, or

* Provide a much stronger methodological justification for treating the scale as a composite indicator, including empirical justification for the cutoff (e.g., median split or ROC analysis) and re-running the main analysis with sensitivity checks.

Without addressing this, the validity of the core findings remains questionable.

Additionally, please report the response rate and add non-response bias to the limitations.

[Note: HTML markup is below. Please do not edit.]

[NOTE: If reviewer comments were submitted as an attachment file, they will be attached to this email and accessible via the submission site. Please log into your account, locate the manuscript record, and check for the action link "View Attachments". If this link does not appear, there are no attachment files.]

To ensure your figures meet our technical requirements, please review our figure guidelines: https://journals.plos.org/plosone/s/figures

You may also use PLOS’s free figure tool, NAAS, to help you prepare publication quality figures: https://journals.plos.org/plosone/s/figures#loc-tools-for-figure-preparation.

NAAS will assess whether your figures meet our technical requirements by comparing each figure against our figure specifications.

# Author comment: Author response to Decision Letter 4

**DOI:** 10.1371/journal.pone.0350860.r008

9 May 2026

We added our comments in Response to Reviewer/ Editor as an attachment.

Attachment Submitted filename: Response to reviewers R5.docx

# Editor report: Decision Letter 4

**DOI:** 10.1371/journal.pone.0350860.r009

**Authors:** Mohamed Said

12 May 2026

PONE-D-25-57473R4

Mental Illness Stigma and its impact on Help-Seeking Behavior among Residents of the Eastern Region, Saudi Arabia: A Cross-Sectional Study

PLOS One

Dear Dr. Al Jowf,

Thank you for submitting your manuscript to PLOS ONE. After careful consideration, we feel that it has merit but does not fully meet PLOS ONE’s publication criteria as it currently stands. Therefore, we invite you to submit a revised version of the manuscript that addresses the points raised during the review process.

Please submit your revised manuscript by Jun 25 2026 11:59PM. If you will need more time than this to complete your revisions, please reply to this message or contact the journal office at plosone@plos.org. When you're ready to submit your revision, log on to https://www.editorialmanager.com/pone/ and select the 'Submissions Needing Revision' folder to locate your manuscript file.

Please include the following items when submitting your revised manuscript:

- A letter that responds to each point raised by the academic editor and reviewer(s). You should upload this letter as a separate file labeled 'Response to Reviewers'.
- A marked-up copy of your manuscript that highlights changes made to the original version. You should upload this as a separate file labeled 'Revised Manuscript with Track Changes'.
- An unmarked version of your revised paper without tracked changes. You should upload this as a separate file labeled 'Manuscript'.

If you would like to make changes to your financial disclosure, please include your updated statement in your cover letter. Guidelines for resubmitting your figure files are available below the reviewer comments at the end of this letter.

If applicable, we recommend that you deposit your laboratory protocols in protocols.io to enhance the reproducibility of your results. Protocols.io assigns your protocol its own identifier (DOI) so that it can be cited independently in the future. For instructions see: https://journals.plos.org/plosone/s/submission-guidelines#loc-laboratory-protocols. Additionally, PLOS ONE offers an option for publishing peer-reviewed Lab Protocol articles, which describe protocols hosted on protocols.io. Read more information on sharing protocols at https://plos.org/protocols?utm_medium=editorial-email&utm_source=authorletters&utm_campaign=protocols.

As the corresponding author, your ORCID iD is verified in the submission system and will appear in the published article. PLOS supports the use of ORCID, and we encourage all coauthors to register for an ORCID iD and use it as well. Please encourage your coauthors to verify their ORCID iD within the submission system before final acceptance, as unverified ORCID iDs will not appear in the published article. *Only*     the individual author can complete the verification step; PLOS staff *cannot*     verify ORCID iDs on behalf of authors.

We look forward to receiving your revised manuscript.

Kind regards,

Mohamed Ahmed Said, Ph.D.

Academic Editor

PLOS One

Journal Requirements:

If the reviewer comments include a recommendation to cite specific previously published works, please review and evaluate these publications to determine whether they are relevant and should be cited. There is no requirement to cite these works unless the editor has indicated otherwise.

Please review your reference list to ensure that it is complete and correct. If you have cited papers that have been retracted, please include the rationale for doing so in the manuscript text, or remove these references and replace them with relevant current references. Any changes to the reference list should be mentioned in the rebuttal letter that accompanies your revised manuscript. If you need to cite a retracted article, indicate the article’s retracted status in the References list and also include a citation and full reference for the retraction notice.

Additional Editor Comments:

Please report the number of eligible individuals who were contacted and the calculated response rate (%). If this information was not recorded, please state this as a limitation and justify why it could not be calculated.

[Note: HTML markup is below. Please do not edit.]

**********

[NOTE: If reviewer comments were submitted as an attachment file, they will be attached to this email and accessible via the submission site. Please log into your account, locate the manuscript record, and check for the action link "View Attachments". If this link does not appear, there are no attachment files.]

To ensure your figures meet our technical requirements, please review our figure guidelines: https://journals.plos.org/plosone/s/figures

You may also use PLOS’s free figure tool, NAAS, to help you prepare publication quality figures: https://journals.plos.org/plosone/s/figures#loc-tools-for-figure-preparation.

NAAS will assess whether your figures meet our technical requirements by comparing each figure against our figure specifications.

# Author comment: Author response to Decision Letter 5

**DOI:** 10.1371/journal.pone.0350860.r010

17 May 2026

Manuscript is updated as suggested by editor .

Attachment Submitted filename: Response to reviewers R6.docx

# Editor report: Decision Letter 5

**DOI:** 10.1371/journal.pone.0350860.r011

**Authors:** Mohamed Said

19 May 2026

Mental Illness Stigma and its impact on Help-Seeking Behavior among Residents of the Eastern Region, Saudi Arabia: A Cross-Sectional Study

PONE-D-25-57473R5

Dear Dr. Ghazi I. Al Jowf,

We’re pleased to inform you that your manuscript has been judged scientifically suitable for publication and will be formally accepted for publication once it meets all outstanding technical requirements.

Within one week, you’ll receive an e-mail detailing the required amendments. When these have been addressed, you’ll receive a formal acceptance letter and your manuscript will be scheduled for publication.

An invoice will be generated when your article is formally accepted. Please note, if your institution has a publishing partnership with PLOS and your article meets the relevant criteria, all or part of your publication costs will be covered. Please make sure your user information is up-to-date by logging into Editorial Manager at Editorial Manager® and clicking the ‘Update My Information' link at the top of the page. For questions related to billing, please contact billing support.

If your institution or institutions have a press office, please notify them about your upcoming paper to help maximize its impact. If they’ll be preparing press materials, please inform our press team as soon as possible -- no later than 48 hours after receiving the formal acceptance. Your manuscript will remain under strict press embargo until 2 pm Eastern Time on the date of publication. For more information, please contact onepress@plos.org.

Kind regards,

Mohamed Ahmed Said, Ph.D.

Academic Editor

PLOS One

Additional Editor Comments (optional):

Reviewers' comments:

# Editor report: Acceptance letter

**DOI:** 10.1371/journal.pone.0350860.r012

**Authors:** Mohamed Said

PONE-D-25-57473R5

PLOS One

Dear Dr. Al Jowf,

I'm pleased to inform you that your manuscript has been deemed suitable for publication in PLOS One. Congratulations! Your manuscript is now being handed over to our production team.

At this stage, our production department will prepare your paper for publication. This includes ensuring the following:

* All references, tables, and figures are properly cited

* All relevant supporting information is included in the manuscript submission,

* There are no issues that prevent the paper from being properly typeset

You will receive further instructions from the production team, including instructions on how to review your proof when it is ready. Please keep in mind that we are working through a large volume of accepted articles, so please give us a few days to review your paper and let you know the next and final steps.

Lastly, if your institution or institutions have a press office, please let them know about your upcoming paper now to help maximize its impact. If they'll be preparing press materials, please inform our press team within the next 48 hours. Your manuscript will remain under strict press embargo until 2 pm Eastern Time on the date of publication. For more information, please contact onepress@plos.org.

You will receive an invoice from PLOS for your publication fee after your manuscript has reached the completed accept phase. If you receive an email requesting payment before acceptance or for any other service, this may be a phishing scheme. Learn how to identify phishing emails and protect your accounts at https://explore.plos.org/phishing.

If we can help with anything else, please email us at customercare@plos.org.

Thank you for submitting your work to PLOS ONE and supporting open access.

Kind regards,

PLOS ONE Editorial Office Staff

on behalf of

Dr. Mohamed Ahmed Said

Academic Editor

PLOS One
